# Openness weighted association studies: leveraging personal genome information to prioritize non-coding variants

**DOI:** 10.1093/bioinformatics/btab514

**Published:** 2021-07-14

**Authors:** Shuang Song, Nayang Shan, Geng Wang, Xiting Yan, Jun S Liu, Lin Hou

**Affiliations:** Center for Statistical Science, Department of Industrial Engineering, Tsinghua University, 100084 Beijing, China; Center for Statistical Science, Department of Industrial Engineering, Tsinghua University, 100084 Beijing, China; University of Queensland Diamantina Institute, University of Queensland, 4102 Brisbane, Australia; Department of Internal Medicine, Section of Pulmonary, Critical Care, and Sleep Medicine, Yale School of Medicine, New Haven, CT 06519, USA; Department of Biostatistics, Yale School of Public Health, New Haven, CT 06510, USA; Department of Statistics, Harvard University, Cambridge, MA 02138, USA; Center for Statistical Science, Department of Industrial Engineering, Tsinghua University, 100084 Beijing, China; MOE Key Laboratory of Bioinformatics, School of Life Sciences, Tsinghua University, 100084 Beijing, China

## Abstract

**Motivation:**

Identification and interpretation of non-coding variations that affect disease risk remain a paramount challenge in genome-wide association studies (GWAS) of complex diseases. Experimental efforts have provided comprehensive annotations of functional elements in the human genome. On the other hand, advances in computational biology, especially machine learning approaches, have facilitated accurate predictions of cell-type-specific functional annotations. Integrating functional annotations with GWAS signals has advanced the understanding of disease mechanisms. In previous studies, functional annotations were treated as static of a genomic region, ignoring potential functional differences imposed by different genotypes across individuals.

**Results:**

We develop a computational approach, Openness Weighted Association Studies (OWAS), to leverage and aggregate predictions of chromosome accessibility in personal genomes for prioritizing GWAS signals. The approach relies on an analytical expression we derived for identifying disease associated genomic segments whose effects in the etiology of complex diseases are evaluated. In extensive simulations and real data analysis, OWAS identifies genes/segments that explain more heritability than existing methods, and has a better replication rate in independent cohorts than GWAS. Moreover, the identified genes/segments show tissue-specific patterns and are enriched in disease relevant pathways. We use rheumatic arthritis and asthma as examples to demonstrate how OWAS can be exploited to provide novel insights on complex diseases.

**Availability and implementation:**

The R package OWAS that implements our method is available at https://github.com/shuangsong0110/OWAS.

**Supplementary information:**

Supplementary data are available at *Bioinformatics* online.

## 1 Introduction

In the past decade, genome-wide association studies (GWAS) have identified tens of thousands of genetic associations (Jostins and Barrett, 2011), which have led to new insights into etiologies of many diseases. Despite the success, how to interpret the functional relevance of detected loci remains a paramount challenge. The difficulties partly lie in that the causal genes mediating variant effects on the trait are rarely ascertainable from GWAS data alone (Wainberg *et al.*, 2019) without external information. Furthermore, the majority of GWAS loci (∼89%) lie within non-coding regions (Gusev *et al.*, 2014). To understand biological mechanisms underlying these significant associations, it is necessary to incorporate function annotations in non-coding regions in the genome (Hou and Zhao, 2013).

Most existing literature assumes that annotated SNPs are more likely to be causal and are enriched for heritability. For example, Chung *et al.* (2014) proposed a statistical approach to prioritize GWAS results by integrating pleiotropy and annotation. Lu *et al.* (2016) established GenoSkyline to integrate tissue-specific functional annotations to improve signal prioritization. DIVAN (Chen *et al.*, 2016) identifies non-coding disease risk variants by integrating multiple genomic features. Watanabe *et al.* (2017) developed a convenient platform FUMA to facilitate functional annotations of GWAS results, gene prioritization and interactive visualization. In addition, transcriptome-wide association studies (TWAS) leverage genetically regulated gene expression information of each individual in the GWAS cohort to aggregate SNP-level effects into gene-level effects, and to further discover gene-trait associations (Gusev *et al.*, 2016). These prioritization approaches provide novel insights into biological mechanism, enabling researchers to better understand gene regulation, as well as the pathogenesis of human diseases.

In this article, we are particularly interested in understanding the role of chromatin accessibility in human complex diseases. Chromatin accessibility is the degree to which nuclear macromolecules are able to physically contact chromatinized DNA (Klemm *et al.*, 2019). As an important epigenetic change, chromatin accessibility is a conserved eukaryotic characteristic of active regulatory elements, including promoters, enhancers, silencers, insulators, transcription factor (TF) binding sites and active histone modifications. The accessible regions, which are also known as open regions, often work together with TFs, RNA polymerases and other cellular machines to regulate gene expression. Interestingly, 57% of the non-coding GWAS hits lie in open chromatin (spanning 42% of the genome), implying that chromatin accessibility will help understand the genetic mechanism of complex diseases (Finucane *et al.*, 2015; Maurano *et al.*, 2012; Ritchie *et al.*, 2014).

Although several high-throughput biotechnologies, such as DNase-seq, FAIRE-seq and ATAC-seq (Min *et al.*, 2017), have been developed to measure chromatin accessibility, experimental measurement in large cohort is costly and not common. Therefore, computational approaches that predict chromatin accessibility from DNA sequences have been proposed. Notably, the method deltaSVM was developed based on gkm-SVM classifier and quantifies cell-type-specific effects of variants on DNase I sensitivity in their native genomic contexts (Lee *et al.*, 2015). A deep learning-based method was proposed for predicting assay-specific epigenetic consequences (Hoffman *et al.*, 2019). DeepCage incorporates cell type specific transcriptome profile to predict regulatory elements (Liu *et al.*, 2021). Accurate *in silico* predictions of chromatin accessibility provide new opportunities for us to understand roles of non-coding variants in disease mechanisms. Some recent work leverages chromatin accessibility information to interpret GWAS variants (Li *et al.*, 2020a; Soskic *et al.*, 2019). Other representative work includes STAAR, which incorporates functional annotations to empower rare variant association analysis (Li *et al.*, 2020b), and GARFIELD, which integrates functional annotation in association models and classifies disease-relevant genomic features to bring novel biological insights (Iotchkova *et al.*, 2019). In addition, one can also leverage predicted regulatory information to split GWAS SNPs into functional units, and identify phenotype associations of SNPs in each functional unit (Arloth *et al.*, 2020). However, existing approaches mainly treat functional annotations as an inherent attribute of a genomic segment, while their variations in personal genomes are ignored. In other words, these approaches assume that the epigenetic status of a genomic segment is homogeneous among all subjects. In fact, epigenetic studies in reference panels have shown a substantial variation in chromatin accessibility across individuals. We hypothesize that incorporating chromatin accessibility prediction in personal genomes in GWAS will further improve our understanding of the roles of non-coding variants in disease etiology.

Here, we develop a systematic framework, Openness Weighted Association Studies (OWAS), which leverages *in silico* predictions of chromatin accessibility in personal genomes to prioritize GWAS SNPs. Individual-level openness, i.e. quantitative measure of chromatin accessibility, is predicted for each genomic segment and the openness scores are used as weights in subsequent association analysis. OWAS can be considered as a post-GWAS prioritization approach that integrates external information to prioritize disease-related genes/segments. Through extensive simulations and real data analyses, we find that OWAS identifies genes/segments that are more interpretable and reliable, and explains more heritability than existing methods. Furthermore, OWAS can take GWAS summary statistics as inputs and therefore does not require individual-level genotype data. Its computational framework can be easily extended to incorporate other epigenetic features. Overall, our results show that integrating functional predictions in personal genomes with GWAS can provide more precise interpretations of roles of non-coding variants in disease mechanism and shed insight on genetic architectures of complex traits.

## 2 Materials and methods

### 2.1 Method overview

**Fig. 1.**
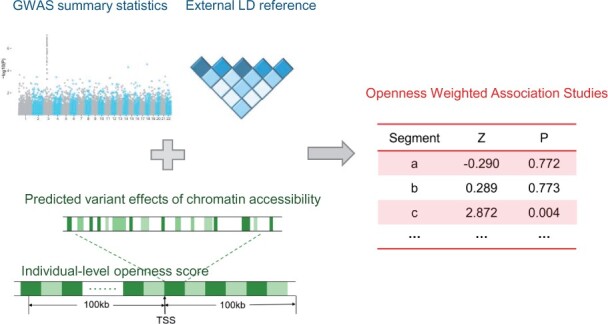
Schematic diagram of OWAS. Cell-type-specific predictions of individual-level chromosome accessibility (green part) and GWAS data (blue part) are integrated via statistical modeling to derive the test statistics of OWAS (red part)

OWAS is a segment-based association approach, in which openness of a genomic segment is predicted for each personal genome, and subsequently tested for the association with the phenotype of interest (Fig. 1). Importantly, we distinguish functions of non-coding genomic elements across different individuals by the genotype of SNPs embedded. In order to accommodate traits/diseases for which only GWAS summary statistics are available, we provide an analytical formula to approximate the OWAS statistics. In particular, we take 100 KB (Supplementary Note S1.1) up and down-stream from the transcription start sites of genes as regulatory regions, which covers most of the regulatory variants (Nasser *et al.*, 2021). We then divide the regions into segments of 5 KB, and calculate an openness score for each segment of each individual in a GWAS cohort. The openness score is a weighted aggregation of genotypes of SNPs in the segment, while the weight of an SNP is predicted by machine learning approaches in the literature.

Let *X* denote the genotype matrix of *n* individuals (rows) and *m* SNPs (columns), and let *X_ij_* be the genotype of the *j*-th SNP of the *i*-th individual. The segment-level openness score $O_{i,s}$ predicted for segment *s* in individual *i* is

(1) $$O_{i,s}=\sum_{j\in \Omega_{s}}w_{j}X_{ij},$$

where *w_j_* is the predicted openness effect for the *j*-th SNP, and Ω_*s*_ indicates the set of SNPs in segment *s*. To study the phenotypic association of each openness score, we posit the following linear phenotype model:

(2) $$Y=\alpha +O_{s}\gamma_{s}+\epsilon ,$$

where γ_*s*_ is called the effect size for segment *s*, and the error *ϵ* follows the normal distribution. The OWAS *Z*-score is simply

(3) $$Z_{s}=\frac{{\hat{\gamma }}_{s}}{\mathrm{se(}{\hat{\gamma }}_{s})}.$$

When individual-level genotype data *X* is provided, *O_s_* can be directly calculated with Equation (1), and OWAS *Z*-scores can be derived by fitting the linear regression model in Equation (2). When only GWAS summary statistics are available, we can approximate the OWAS *Z*-score as:

(4) $$Z_{s}=\frac{{\hat{\gamma }}_{s}}{\mathrm{se}({\hat{\gamma }}_{s})}\approx \sum_{j\in \Omega_{s}}w_{j}\frac{{\hat{\sigma }}_{j}}{{\hat{\sigma }}_{s}}z_{j},$$

where *z_j_* is the *z*-score for the *j*-th SNP in GWAS summary statistics; σ^j2 is the sample variance of SNP *j* and σ^s2 is sample variance of the openness scores in segment *s*. The derivation of Equation (4) is provided in Supplementary Note S1.2.

### 2.2 Prediction of regulatory effect in personal genomes

We derive the predicted openness effect (*w_j_*) of each SNP with deltaSVM (Lee *et al.*, 2015), which is a sequence-based computational method that predicts allelic regulatory effects of SNPs with cell-type specificity. We trained the model on DNase I-hypersensitive sites (DHSs) of 12 common cell types from the UW ENCODE Project (http://www.beerlab.org/deltasvm/). A list of the cell types is provided in Supplementary Table S1.

### 2.3 Cell type selection

OWAS synthesizes openness scores trained from distinct cell types. We provide a strategy to automatically select a relevant cell type by GARFIELD (Iotchkova *et al.*, 2019), which identifies the cell type of which open chromatin marks are most enriched for trait-specific GWAS signals. The selected cell type is then used for downstream analyses.

### 2.4 Simulations

We first simulated GWAS *z*-scores. Associated SNPs were divided into three disjoint groups, *C*_1_, *C*_2_ and *C*_3_, with high, medium and low heritability, respectively, according to their ranks of openness scores. For an associated SNP in *C_k_*, its effect size was simulated from a mean-zero normal distribution with variance τ_*k*_. Let *C*_0_ denote the set of unassociated SNPs, with proportion fixed at 0.9. Then we have $\tau_{k}=h^{2}(C_{k})/M(C_{k})$, where $M(C_{k})$ is the number of SNPs in group *C_k_*. We further simulated SNP-level *z*-scores according to:

(5) $$z|R,\beta ∼N(\sqrt{n}R\beta ,R),$$

where β is the vector of SNP-level effect sizes, and ***R*** is the linkage disqeuilibrium (LD) matrix, which is estimated from 1000 Genomes Project European samples on chromosome 22. The sample size *n* was fixed at 10 000. The number of SNPs is 141 123. We set $h^{2}(C_{k})$ to be 50%, 20% and 10% for *k *=* *1, 2, 3, which contain 0.5%, 1.5% and 8% of the total number of SNPs, respectively.

As a comparison, we used an unweighted model regarding all SNPs in each segment equally. We compared the performance of OWAS with the unweighted model, by ranking the segments with the derived *P*-values and estimating the heritability explained by specific proportions of prioritized segments. The simulations were repeated for 100 times.

### 2.5 Pathway enrichment analysis

We tested the enrichment of OWAS genes in KEGG pathways (Kanehisa *et al.*, 2008) using the R package ‘clusterProfiler’ (Yu *et al.*, 2012). For multiple testing error control, we used the Benjamini–Hochberg procedure to control the false discovery rate (FDR) at level 0.05 (Benjamini and Hochberg, 1995).

### 2.6 Compared methods

FUSION (Gusev *et al.*, 2016) is a TWAS method that studies transcriptome-phenotype associations. For FUSION-open, we extracted genes that have overlaps with the DNase-seq peaks in the corresponding cell type, and used FUSION to prioritized those genes. The CADD scores (Rentzsch *et al.*, 2019) supports prioritization of non-coding variants by integrating a range of annotations. We annotated the variant CADD scores by FUMA (Watanabe *et al.*, 2017) and ranked the SNPs accordingly, which is denoted by FUMA-CADD.

### 2.7 Materials

A detailed description of the GWAS datasets, the chromatin accessibility data and TWAS results is provided in Supplementary Materials.

## 3 Results

### 3.1 OWAS segments explain more heritability

For real data experiments, we applied OWAS to three complex traits including Crohn’s disease (CD), hypertension (HT) and RA. The cell types selected for each trait is provided in Supplementary Table S2. In order to understand the influence of cell types, we applied OWAS with 12 common cell types from UW ENCODE Project and compared the prioritization results of each cell type to the selected cell type. OWAS identified most disease-relevant genes (Supplementary Note S1.3 and Supplementary Table S3), possibly due to the shared regulatory mechanism between cell types. Meanwhile, OWAS retains its superiority in explained heritability compared to other methods, even when the cell type is not optimal for the trait (Supplementary Fig. S1).

**Fig. 2.**
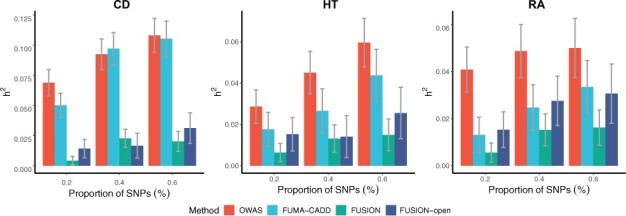
OWAS segments are more enriched for heritability compared to the FUSION genes, FUSION genes in open regions and SNPs prioritized by CADD scores annotated by FUMA, on CD, HT and rheumatic arthritis (RA). OWAS segments and the FUSION genes were ranked by their *P*-values, respectively, and the proportion of SNPs (x-axis) and the explained heritability (y-axis) at varying cutoffs are displayed. The error bar corresponds to the standard error of the heritability estimated by GCTA software. The discovery cohorts are derived using UKB summary statistics and the heritability was estimated with the WTCCC individual-level genotype data

As a comparison, we also prioritized genes/SNPs using FUSION, FUSION-open and FUMA-CADD (Section 2.6). The UK Biobank (UKB) summary statistics were used as the discovery cohort, and all the methods were evaluated with the WTCCC datasets (Consortium *et al.*, 2007), which are independent from the discovery cohorts. We excluded the HLA region (chr6:28,477,797-33,448,354, hg19) in the heritability analysis due to the unusual LD structure and genetic architecture. For the compared methods, we ranked the SNPs/segments/genes according to their significance levels, and estimated the heritability explained by prioritized SNPs using the GCTA software (Yang *et al.*, 2011). To avoid comparisons at arbitrary cutoffs, we varied the significance thresholds for all the methods, and calculated the proportion of SNPs and the explained heritability at each threshold (Fig. 2). With the same proportion of SNPs, OWAS segments explain more heritability compared to other methods, and the patterns are consistent across the three different traits. We also notice that the FUSION genes within the open chromatin regions are more enriched for heritability than those outside, which highlights that the integration of chromatin accessible information improves the heritability enrichment.

The improvement in explained heritability is also validated in simulations. We simulated GWAS *z*-scores from a genetic model with heritability enriched in SNPs with high openness effects (Section 2.4). The heritability of genomic segments detected by OWAS is significantly greater than that of the unweighted method (Supplementary Fig. S2).

### 3.2 OWAS prioritized SNPs have higher replication rate

**Fig. 3.**
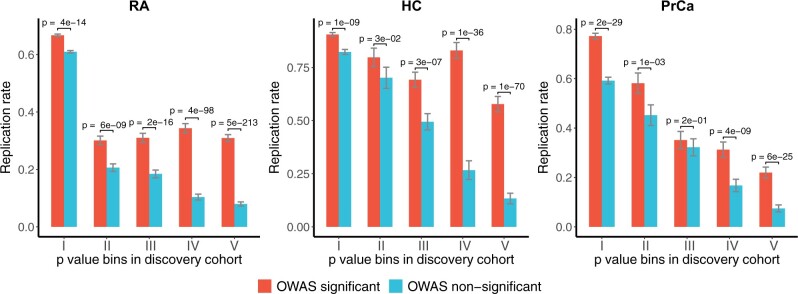
Replication of OWAS results. OWAS was performed with GWAS summary statistics from both the discovery cohort (with larger sample sizes) and the replication cohort on rheumatoid arthritis (RA), HC and PrCa. In the discovery cohort, GWAS SNPs were divided into 5 bins according to their *P*-values [I: (0, 1e–06), II: [1e–06, 1e–05), III: [1e–05, 1e–04), IV: [1e–04, 1e–03), V: [1e–03, 1e–02)]. In the replication cohort, GWAS significant SNPs were identified with a relaxed threshold (*P *<* *5e–02). In each bin, the SNPs were broken down into prioritized and not prioritized groups by the OWAS results (*P *<* *5e–08). The *P*-values were derived from the binomial test

We evaluated the replication rate of SNPs prioritized by the OWAS analysis in independent cohorts. For RA and prostate cancer (PrCa), we took the largest meta-analysis available as the discovery cohort and the UKB studies as the replication cohort. For high cholesterol (HC), we took UKB studies as the discovery cohort due to their large sample sizes (*n *=* *361 141), and used the study by Teslovich *et al.* (2010) for the total cholesterol (*n *=* *100 184) as the replication cohort. For each trait, we used a two-stage process. We first binned the SNPs by their GWAS *P*-values in the discovery cohort. Then, in each bin, we compared the replication rate of prioritized SNPs (i.e. SNPs harbored by significant segments in OWAS) to that of the other SNPs. OWAS prioritized SNPs had a greater replication rate, and the trend was consistent across different traits and different *P*-value bins (Fig. 3). The results indicate that OWAS effectively identifies truly associated SNPs in SNPs with moderate *P*-values, and the effect is more prominent for less significant SNPs.

We further compared the replicability of OWAS with that of DeepWAS (Arloth *et al.*, 2020) (Supplementary Note S1.4), a recently developed method that identifies genotype-phenotype associations by incorporating predicted regulatory information. DeepWAS requires individual-level genotype data for model training, thus we used the imputed WTCCC data as the discovery cohort and the UKB data for replication. OWAS segments achieved higher replication rates in most scenarios compared with DeepWAS (Supplementary Fig. S3).

### 3.3 Functional analysis of OWAS segments illustrates disease mechanism

**Fig. 4.**
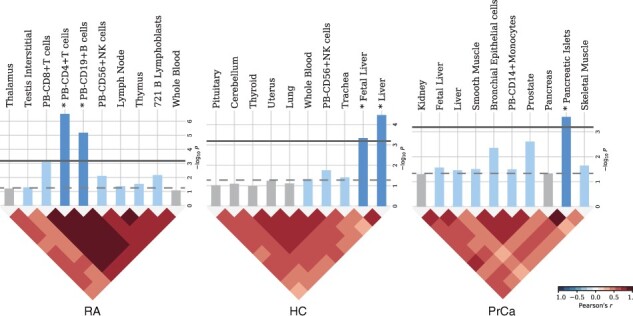
SNPsea analysis on OWAS identified segments. Empirical *P*-values for the enrichment of tissue-specific expression in profiles of 79 human tissues and cells (Gene Atlas), with the top 10 shown. Horizontal dashed and solid lines show *P*-value cutoffs at the 0.05 level (unadjusted and with Bonferroni correction). The heat maps show the Pearson correlation coefficients for pairs of expression profiles ordered by hierarchical clustering with UPGMA

We identified genes tagged by significant OWAS segments, and examined their tissue specificity and functional enrichment. In particular, OWAS identified 398, 313 and 211 genes with 4895, 1635 and 706 segments associated with RA, HC and PrCa, respectively. We performed SNPsea analysis (Slowikowski *et al.*, 2014) for segments identified by OWAS for each trait in order to quantify the enrichment of tissue-specific expressions (Fig. 4). OWAS segments for RA marked genes specifically expressed in CD4+ T cells (*P *=* *3.0e–07) and CD19+ B cells (*P *=* *7.0e–06), and these two cell types are both associated with autoimmune diseases (Konya *et al.*, 2009; Suzuki *et al.*, 2008). Specifically, CD4+ T cells, including T helper (Th) and regulatory T (Treg) cells, play critical roles in pathogenesis of RA (Kondo *et al.*, 2018). In addition, increasing evidence suggests that B cells, which exclusively express CD19, participate in the pathogenesis of RA including autoantibody production and CD4+ T cell activation. Pre-clinical studies also propose CD19 as a promising therapeutic target for RA (Tedder, 2009). Similarly, our OWAS analyses on HC identified liver as the tissue with the most significant enrichment (*P *=* *3.4e–05) for cell-type-specific gene expression relative to 78 other tissues in the Gene Atlas (Su *et al.*, 2004). The pancreatic islets were identified as the most enriched (*P *=* *2.5e–04) tissue for PrCa, and the prostate came second (*P *=* *2.5e–03).

We further investigated overlaps between OWAS segments and predicted chromatin states from the 15-state ChromHMM model (Ernst and Kellis, 2017). While OWAS segments are generally enriched in open chromatin regions, we still observe an enrichment of active chromatin states in PrCa (P< 0.05 with Bonferroni correction) when the bias toward DNase-seq peaks is accounted for through a permutation test (Supplementary Note S1.5 and Supplementary Fig. S4).

### 3.4 OWAS results in RA and ATH provide novel insights into the disease etiology

**Fig. 5.**
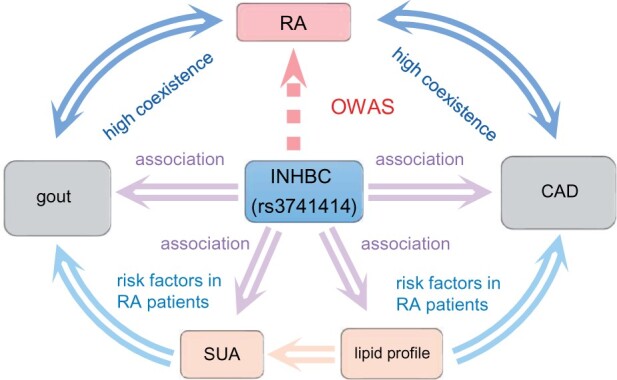
Putative mechanism for RA, CMDs and gout. The connections confirmed by previous studies are linked with solid lines. OWAS identified the *INHBC* as the RA associated locus, validating the common genetic mechanism between CMDs and RA

We highlight two examples on RA and arthritis and asthma (ATH) to show how OWAS can provide novel insights into the disease etiology. We mapped the significant segments OWAS identified to the closest genes, which we simply refer to as the OWAS genes. For RA, 398 genes were identified by OWAS at the 0.05 significance level after the Bonferroni correction, among which 205 were outside HLA regions. The large number of OWAS genes located in HLA regions (48.49%) validates the significant role of HLA regions in the immune system. In fact, strong associations between HLA regions and autoimmune diseases have been well documented in the literature (Simmonds and Gough, 2007), thus we focus here on non-HLA genes in the discussion. The top 10 OWAS genes for RA are listed in Supplementary Table S4, among which *PHTF1, HIPK1, PTPN22* and *RSBN1* have been reported in GWAS of RA. Although not directly mapped to RA, *AP4B1* and *BCL2L15* are located in RA-associated locus 1p13.2, and were reported to have interactions with enhancers (Gao and Qian, 2019). *LAMA3* has been previously associated with reticulocyte count, a blood biomarker for hematologic abnormalities, like anemia (Astle *et al.*, 2016), which is one of the most frequent extra-articular organ manifestations in RA (Komrokji *et al.*, 2016). *OLFML3* serves as both a scaffold protein that recruits bone morphogenetic protein (BMP) to its substrate chordin in Xenopus (Inomata *et al.*, 2008). BMPs have been demonstrated playing a key part in destructive and remodeling arthritis (Lories and Luyten, 2007). *OLFML3* also acts as a vascular tissue remodeler with pro-angiogenic properties by modulating critical signaling circuits such as Notch pathways (Tomarev and Nakaya, 2009), which underlies inflammation and pathology in RA (Wei *et al.*, 2020). In addition, we provide a potential explanation that there exists common pathogenesis or causal relationship between cardiometabolic diseases (CMD) and RA with common genetic factors regulated by *INHBC* (Fig. 5). There have been studies revealing the connection between RA, CMD, serum uric acid (SUA) (Tin *et al.*, 2019) and lipid profile including triglyceride (TG), high density lipoprotein cholesterol (Hoffmann *et al.*, 2018) and *INHBC*. For example, RA has long been associated with coronary heart disease and gout, which has been strongly linked to CMD (Picavet and Hazes, 2003), and increasing prevalence and rate of progression of atherosclerosis. The *INHBC* has been previously associated with SUA and lipid profile (e.g. TG) (Gorski *et al.*, 2017; Hoffmann *et al.*, 2018). A GWAS of uric acid in healthy controls, RA patients has revealed probable association between SNP rs3741414 *INHBC* and SUA level (Son *et al.*, 2014). A recently developed latent causal variable model also showed that lipid related traits have partially causal effects on SUA (Tin *et al.*, 2019). In our study, we identified *INHBC* as an OWAS gene for RA, validating the common genetic mechanism between CMD and RA.

We also examined the enrichment of OWAS genes identified for RA in KEGG pathways. We found 16 pathways were significantly enriched (FDR < 0.05, Supplementary Fig. S5), including the RA pathway, T cell receptor signaling, NF-κB signaling and JAK-STAT signaling pathways, which are known RA-related pathways. Notably, NF-*κ*B (Potter *et al.*, 2010) and JAK-STAT (McInnes and Schett, 2017) signaling pathways have been translated into therapeutic use (e.g. TNF-α antagonist and JAK inhibitor). Besides, toxoplasmosis has been previously associated with autoimmune diseases (Shapira *et al.*, 2012). Some infection related pathways are enriched, such as measles, human papillomavirus infection and hepatitis B pathways. RA is known to be associated with an increased risk of serious infection (Cobb *et al.*, 1953). The elevated infection susceptibility of patients with RA is conceivably due to premature aging of the immune system, as RA contributes to weakened protection against infectious organisms, chronic comorbid conditions, as well as sequelae of immunosuppressive treatment (Listing *et al.*, 2013). The analysis has highlighted important roles of genes in infection related pathways in RA, including *AKT3, ATF6B, ATP6V1F, ATP6V1G2, BAK1, CD28, CD40, CDK2, CSNK2B, FADD, FOXO1, HES5, HSPA1A, HSPA1B, HSPA1L, ICAM1, IL2, LAMA3, NOTCH4, PSMD3, STAT1, STAT4, TAB1, TAP1, TAP2, TAPBP, TNF, TYK2* and *TNXB*, suggesting that the elevated risk of infection in RA patients can be explained by the genetic inherence and pathobiology of the disease itself, independent of immunosuppressive treatment (Listing *et al.*, 2013).

We use RA as an example to compare OWAS, TWAS and CAVIAR (Hormozdiari *et al.*, 2014), a statistical fine-mapping method. Although the three methods differ in principle (Supplementary Note S1.6), there are some overlaps in the significant genes identified (Supplementary Fig. S6). We further performed pathway enrichment analysis in the common and exclusive genes to compare the methods (Supplementary Fig. S7). We found that five of the eight pathways discussed previously were still significantly enriched after removing the CAVIAR genes or FUSION genes from the OWAS genes. In contrast, no enrichment was found in the common genes, which are defined as genes identified by more than two methods, indicating that OWAS identifies unique association signals compared to CAVIAR and FUSION.

In parallel to RA studies, we applied OWAS to analyze UKB ATH summary statistics (Supplementary Note S1.7 and Supplementary Figs. S8 and S9). We identified a cluster of genes in 17q21, within which increased expressions of *ORMDL3* and *GSDMB* lead to an increased airway hyper-reactivity, which is the characteristic of ATH and also validated by *in vivo* studies (Miller *et al.*, 2018). Further, we give a potential biological mechanism between *LRRC32* and ATH, which underscores the capability of OWAS to interpret epigenetic mechanisms of non-coding variants in risk loci.

### 3.5 Predicted openness scores

DNase I hypersensitivity and histone modifications mark regulatory elements and regions of active transcription (Heintzman *et al.*, 2009; Wang *et al.*, 2008). Here, we show that the predicted openness scores vary significantly among active chromatin regions. The openness was trained on DHSs from GM12878 cell types (Consortium *et al.*, 2012) by deltaSVM (Lee *et al.*, 2015). We collected publicly available data for three histone modifications that have been previously associated with active promoters and enhancers (Heintzman *et al.*, 2009; Wang *et al.*, 2008), including H3K4me1, H3K4me3 and H3K27ac, in HapMap lymphoblastoid cell lines.

We selected thresholds at 5, 10 and 15, respectively, to define the experimental peak signals and calculated the average of predicted openness scores for variants located in the selected peaks. For comparison, the same number of variants was randomly sampled from the genome and the corresponding openness scores were similarly derived. We ran the sampling process 10 000 times and show the results in Supplementary Figure S10. The averages of predicted openness increased when more stringent thresholds were chosen. For all three histone modifications, the predicted openness in peak regions differed significantly from the background.

### 3.6 Type I error control

In order to evaluate the type I error of our approach, we conducted simulations based on individual-level genotypes from the WTCCC cohort. In particular, we used samples of the type II diabetes study, including 1895 cases and 2872 controls after quality control. Genotype data of chromosome 1 were used for simulation, covering 23 601 SNPs. We split the variants into segments of 5, 20 and 50 SNPs. Openness scores were simulated from a normal distribution. We sampled 100 segments and ran 1000 simulations for each segment. The type I error rates are well controlled, as summarized in Supplementary Table S5.

### 3.7 Sensitivity analysis of segment length

We tested OWAS with segment length fixed at 2.5 KB, 5 KB and 10 KB. Besides, we allowed for variable segment length while fixing the number of SNPs harbored in each segment at 20 (about the median number of SNPs of 5 KB region). The advantages of OWAS in replication rate and heritability enrichment in various traits are retained in all four settings, compared to existing approaches (Supplementary Figs. S11–14). Thus, OWAS results are robust to segment length, and we use 5 KB as the default setting.

## 4 Discussion

Recently, there is a growing interest in understanding roles of non-coding variations in complex human diseases, empowered by the huge amount of data generated from epigenomic profiling efforts and the large scale GWAS of various phenotypes. Here, we develop a computational approach, OWAS, which integrates chromatin accessibility information in association tests. Our approach leverages predicted openness scores trained with machine learning methods, overcoming the difficulties in acquiring individual-specific openness measurement in large cohorts. We also derive a mathematical expression to compute OWAS results without individual-level genotype data, which broadens the scope of its application.

We recognize that recent progress in caQTLs (Gate *et al.*, 2018; Kumasaka *et al.*, 2016) is closely related to our method, as the overlap of caQTLs and GWAS loci can provide insights into how natural genetic variants modulate cis-regulatory elements, in isolation or in concert, to influence complex traits. However, experimental measurement of chromatin accessibility in large cohort is costly and therefore not common. Thus, the power to identify caQTLs is still limited. A comparison between OWAS and caQTLs in provided in Supplementary Note S1.8.

Our method has several limitations. First, the results are influenced by the prediction accuracy of the machine learning methods. Nevertheless, with increased sample sizes and improvements in methodologies, we expect the prediction models to be further improved over the time. Second, when used with GWAS summary statistics, the method requires LD information estimated from a reference panel as input. Therefore, a reference cohort that accurately matches the target cohort is of great significance. Third, although incorporating chromatin accessibility information leads to interesting biological interpretations, we emphasize that the method alone cannot infer causality. Fourth, our method relies on pre-trained cell-type-specific sequence models, and its power could be limited by data availability of relevant cell types. A recent study (Boix *et al.*, 2021) has integrated epigenetic maps from 833 biosamples, containing 733 DNase-seq experiments, showcasing that current epigenome sequencing resources have sufficiently covered many complex traits. Plus, convenient tools [such as openAnnotate (Chen *et al.*, 2021)] can facilitate efficient exploration of openness data across cell types. We expect the power of OWAS to be further enhanced when more data become available.

In conclusion, we have proposed a new segment-based computational framework, OWAS, which leverages and aggregates the prediction of chromosome accessibility in personal genomes to prioritize GWAS signals. In extensive simulations and real data analyses, OWAS identifies genes/segments that explain more heritability than TWAS methods and have high replication rates in independent cohorts. Our method requires only GWAS summary statistics and a reference LD panel, which guarantees its general applicability without any privacy concerns. As sample sizes of GWAS studies continue to grow, directly using summary statistics also helps maintain the computational burden at a constant level and increases the power of the methods. Furthermore, the significant associations identified by OWAS can lead to interesting biological interpretations, as exemplified in real data analyses of RA and ATH.

## Supplementary Material

btab514_Supplementary_Data
