# Supplementary material for: Openness weighted association studies: leveraging personal genome information to prioritize non-coding variants
Source: Bioinformatics. 2021 Jul 14;37(24):4737–43. doi: 10.1093/bioinformatics/btab514 (PMC8665759; doi:10.1093/bioinformatics/btab514)
Supplement: btab514_Supplementary_Data [file btab514_supplementary_data.pdf]

Supplement to "Openness Weighted Association Studies: Leveraging Personal Genome Information to Prioritize Noncoding Variants"

Shuang Song, Nayang Shan, Geng Wang, Xiting Yan, Jun S Liu, Lin Hou

Contents

1 Supplementary Notes **2**
1.1 Definition of regulatory regions 2
1.2 Theoretical derivation of OWAS Z-score 2
1.3 The impact of cell type selection 3
1.4 Comparison between OWAS and DeepWAS 4
1.5 Enrichment analysis of OWAS segments in predicted chromatin states 4
1.6 Theoretical and quantitative discussions of OWAS, TWAS, and statistical fine-mapping approaches 5
1.7 OWAS results in asthma 5
1.8 Comparison between OWAS segments and caQTLs 6

2 Supplementary Materials **7**
2.1 GWAS datasets 7
2.2 Chromatin accessibility data 7
2.3 TWAS results 7

3 Supplementary Tables **8**

4 Supplementary Figures **11**

1 Supplementary Notes

1.1 Definition of regulatory regions

We took 100 KB up and down-stream from the transcription start sites (TSS) of genes as regulatory regions. We note that more variants/segments can be included in the study if a longer pad is used. However, a longer pad also includes more candidate regions, and increase the multiple test burden. Meanwhile, Nasser et al. (2021) [25] have recently shown that the median and mean distance from the noncoding variant to the TSS of the predicted target gene are 13 KB and 63 KB respectively. Thus, 100 KB should sufficiently cover a majority of functional variants.

In addition, we have applied OWAS in the whole genome for the RA GWAS dataset, and the results are displayed in Table S6.

The majority of significant hits ($> 90\%$) are within 100 KB up and down-stream of known TSSs. Fisher's exact test confirms the significant enrichment of OWAS segments in genomic regions of 100 KB of TSSs ($p < 2.2e-16$). Therefore, we consider 100 KB a proper choice for defining candidate regions. In our software, we have added an option of "whole-genome", which allows users to run OWAS in the whole genome, when it is necessary.

1.2 Theoretical derivation of OWAS Z-score

We derive an analytic expression to calculate OWAS Z-scores in Eqn (4) when only GWAS summary statistics are available. In GWAS, we model the phenotype as linear functions of genotype X_j,

$$Y = \lambda + X_j \beta_j + \eta, \tag{1}$$

where λ is the intercept, η is the normally distributed error term, and β_j is the effect size for SNP j. We denote the estimator of marginal effect size of SNP j as $\hat{\beta}_j$, and we have

$$\hat{\beta}_j = \frac{\widehat{cov}(X_j, Y)}{\hat{\sigma}_j^2}, \tag{2}$$

then

$$\widehat{cov}(X_j, Y) = \hat{\beta}_j \hat{\sigma}_j^2, \tag{3}$$

where $\hat{\sigma}_j^2$ is the sample variance of SNP j. We denote the estimator of γ_s as $\hat{\gamma}_s$, which can be calculated by

$$\begin{aligned} \hat{\gamma}_s = \frac{\widehat{cov}(O_s, Y)}{\hat{\sigma}_s^2} &= \frac{\widehat{cov}(\sum_{j \in \Omega_s} w_j X_j, Y)}{\hat{\sigma}_s^2} \\ = \frac{\sum_{j \in \Omega_s} w_j \widehat{cov}(X_j, Y)}{\hat{\sigma}_s^2} &= \frac{\sum_{j \in \Omega_s} w_j \hat{\beta}_j \hat{\sigma}_j^2}{\hat{\sigma}_s^2}, \end{aligned} \tag{4}$$

where w_j is the predicted openness effect for the j-th SNP; Ω_s indicates the set of SNPs in segment s; and $\hat{\sigma}_s^2$ is sample variance of the openness scores in segment s. We denote the vector of openness effect for segment s as $\mathbf{W_s} = (w_{1s}, \cdots, w_{ms})$, where $w_{js} = w_j$ when $j \in \Omega_s$, and $w_{js} = 0$, otherwise. Then we have

$$\hat{\sigma}_s^2 = \widehat{Var}(\sum_{j=1}^{m} w_{js} X_j) = \widehat{Var}(\mathbf{W}_s \mathbf{X_s}) = \mathbf{W}_s^T \widehat{Var}(\mathbf{X_s}) \mathbf{W_s}. \tag{5}$$

We denote R_s^2 and R_j^2 as the proportion of variance explained by the covariates O_s and X_j, and we list the properties:

$$R_s^2 = \hat{\gamma}_s^2 \frac{\hat{\sigma}_s^2}{\hat{\sigma}_Y^2}, \tag{6}$$

and

$$R_j^2 = \hat{\gamma}_j^2 \frac{\hat{\sigma}_j^2}{\hat{\sigma}_Y^2}. \tag{7}$$

Meanwhile,

$$se(\hat{\gamma}_s) = \sqrt{Var(\hat{\gamma}_s)} = \frac{\sigma_\epsilon}{\sqrt{n}\sigma_s} = \frac{\hat{\sigma}_Y \sqrt{1 - R_s^2}}{\sqrt{n}\hat{\sigma}_s}, \tag{8}$$

and

$$se(\hat{\beta}_j) = \sqrt{Var(\hat{\beta}_j)} = \frac{\sigma_\eta}{\sqrt{n}\sigma_j} = \frac{\hat{\sigma}_Y \sqrt{1 - R_j^2}}{\sqrt{n}\hat{\sigma}_j}. \tag{9}$$

Combining Eqn (4), (5), (8), (9), we have

$$\begin{aligned} Z_s = \frac{\hat{\gamma}_s}{se(\hat{\gamma}_s)} &= \sum_{j \in \Omega_s} w_{js} \frac{\hat{\sigma}_j}{\hat{\sigma}_s} \frac{\hat{\beta}_j}{se(\hat{\beta}_j)} \sqrt{\frac{1 - R_j^2}{1 - R_s^2}} \\ &= \sum_{j \in \Omega_s} w_{js} \frac{\hat{\sigma}_j}{\hat{\sigma}_s} z_j \sqrt{\frac{1 - R_j^2}{1 - R_s^2}} \approx \sum_{j \in \Omega_s} w_{js} \frac{\hat{\sigma}_j}{\hat{\sigma}_s} z_j, \end{aligned} \tag{10}$$

where $z_j = \frac{\hat{\beta}_j}{se(\hat{\beta}_j)}$ is the marginal z-score for the j-th SNP, which can be directly obtained from GWAS summary statistics. The $\hat{\beta}_j$ is the OLS estimate of the coefficient in the corresponding marginal linear regression. Here we assume that $\frac{1-R_j^2}{1-R_s^2} \approx 1$, which does not affect the ability to identify the association for most practical purposes, based on the discussion in Barbeira et al. (2018) [4].

1.3 The impact of cell type selection

We used four ways to explore the extent OWAS is influenced by pre-trained models from distinct cell types, including a) similarities among OWAS significant segments and genes, b) ranks of prioritized segments and genes, c) the replicability of OWAS identified segments, and d) enrichments for heritability of OWAS identified segments.

We applied OWAS with 12 common cell types from the UW ENCODE Project (Table S1), with pre-trained deltaSVM models on DNase I–hypersensitive sites (DHS) regions. The prioritization results of each cell type were compared to that of the GM12878 cell line. We use the Jaccard Index and Spearman's rank correlation coefficient to evaluate the similarities between significant segments/genes identified with different cell types (Table S3). We take RA as an example, and the average of Jaccard Index among OWAS segments and genes are 0.474 (sd=0.019) and 0.714 (sd=0.023). As for Spearman's rank correlation, the average values are 0.677 (sd=0.028) and 0.877 (sd=0.012) for identified segments and genes, respectively. Moreover, for all cell types, OWAS prioritized genes retain superiority in replication rate (Figures

S16-S18). For most cell types, the explained heritability of OWAS segments is greater or comparable to existing methods, and several cell lines perform consistently better across different thresholds, namely, GM12878, HepG2, HUVEC, and LNCaP (Figure S1), demonstrating the effectiveness of OWAS when implemented with suboptimal cell types. OWAS can identify most disease-relevant genes when the cell type is not optimal for the trait, due to the shared regulatory mechanism between cell types.

In practice, selections of cell-types can be a problem. To facilitate automatic selections of relevant cell types for OWAS, we provide a strategy based on an existing method, GARFIELD [17], which identifies relevant cell types for a trait by integrating GWAS summary statistics and the cell type specific chromatin features. For each trait, we use GARFIELD to identify the most enriched cell type in open chromatin marks, and then use the selected cell type for OWAS analyses. The cell types selected for the traits analyzed are listed in Table S2.

The cell type selection module is incorporated in our software, but users can opt for user-specified cell-types if there is a strong prior knowledge about them.

1.4 Comparison between OWAS and DeepWAS

DeepWAS [2] is based on individual-level genotype data. Due to data availability, we compared our method with DeepWAS for the WTCCC datasets for CD, HT, and RA. We used the imputed WTCCC individual-level data as the discovery cohort, and tried to evaluate heritability in the UK Biobank summary statistics with stratified LDSC. However, DeepWAS only detected 117, 102, and 103 SNPs in CD, HT, and RA, possibly due to the limited sample size in the WTCCC cohorts (about 4,000 individuals for each trait) and stratified LDSC failed to provide precise estimates of heritability for such small SNP sets. The estimated heritability for the prioritized SNP set for all three traits are close to zero with very large standard errors, and some even have negative estimates. Therefore, we decided to compare OWAS and DeepWAS by replication rate in independent cohorts (Figure S3). OWAS outperformed DeepWAS in most cases, especially with stringent p-value thresholds. We also note that OWAS is compatible with GWAS summary statistics (without the need of individual-level data), which has an advantage over DeepWAS, as the need of individual-level genotypes greatly limits its application. Furthermore, OWAS is more powerful in identifying disease associated SNPs. For example, in RA, OWAS identified 2,449 SNPs with GWAS p-values less than $1e-04$, while obtaining a replication rate of 93.67%. In contrast, DeepWAS only identified 1 SNPs in this group, with no SNPs replicated.

1.5 Enrichment analysis of OWAS segments in predicted chromatin states

We investigated the overlap between OWAS segments and predicted chromatin states from the 15-state ChromHMM model [10]. It is not surprising that the OWAS identified segments were enriched in transcriptional regions compared to depletion in quiescent regions, which may be explained by the bias toward DNase-seq peaks. To address this problem, we performed a permutation test to re-evaluate the enrichment of OWAS segments in different chromatin features. We permute the openness scores inside and outside the DNase-seq peaks of the corresponding cell type separately. In each permutation sample, the enrichment ratio of OWAS segments in each chromatin state is calculated. The permutation was repeated for 5,000 times to derive the null distribution and the subsequent significance of the enrichment results. In this way,

the distribution of openness score is unchanged inside and outside DNase-seq peaks, so the bias toward DNase-seq peaks is accounted for. The enrichment results are shown in Figure S4. The enrichment of active chromatin states is still observed for prostate cancer ($p < 0.05$ with the Bonferroni correction). This provides a novel understanding of functional regulatory mechanisms among complex diseases.

1.6 Theoretical and quantitative discussions of OWAS, TWAS, and statistical fine-mapping approaches

First, the statistical fine mapping methods were developed to pinpoint the causal SNPs from association signals [29], which is different from the goal of OWAS. We prioritize genomic segments in order to identify a subset of SNPs that enrich for GWAS signal, which are not necessarily causal. Second, existing statistical fine mapping approaches treat functional annotations as an inherent attribute of a genomic segment, and variations in personal genomes are not accommodated. In other words, these approaches assume the epigenetic status of a genomic segment is homogeneous among all subjects. In fact, epigenetic studies in reference panels have shown a substantial difference in chromatin accessibility across individuals [13]. We hypothesize that incorporating openness prediction in personal genomes in GWAS will further improve understanding of the roles of noncoding variants in disease etiology. For TWAS methods such as FUSION [15] and PrediXcan [12], they are still gene centric methods, as they focus on gene expression and eQTLs. The vast majority of the noncoding genome is excluded from the analysis. OWAS particularly aims at the prioritization of noncoding association signals.

Quantitively, we used RA as an example to compare OWAS with a fine mapped GWAS method CAVIAR [16] and a TWAS method FUSION. We mapped GWAS SNPs with the nearest gene TSS using a web server for SNP annotation, SNPsnap [26]. Figure S6 shows the overlap among significant genes identified by OWAS, CAVIAR, and FUSION, and OWAS genes were significantly enriched with significant genes in CAVIAR and FUSION.

For functional characterizations, we performed pathway analysis on these common and exclusive SNPs of the three methods on RA analysis. We focused on the eight pathways discussed in Section 3.4 that are directly or indirectly related with RA (Figure S7). Among the eight pathways, T cell receptor signaling, NF-κB signaling, and JAK-STAT signaling pathways are known RA-related pathways. Toxoplasmosis has been previously associated with autoimmune diseases, and the other three pathways are infection related pathways. We found that five of the eight pathways are still significantly enriched after removing the CAVIAR genes or FUSION genes from the OWAS genes, indicating that OWAS identifies signals that are missed in CAVIAR and FUSION studies. We also performed pathway enrichment analysis on common genes, which is defined by genes identified by more than two methods, and we found none of the eight pathways are enriched in the common genes. This may result from the limited genes identified by CAVIAR and FUSION.

1.7 OWAS results in asthma

We applied OWAS to analyze UKB asthma (ATH) summary statistics and identified 3,080 segments along with 516 genes significantly associated with ATH. Among the 516 OWAS genes, 357 were outside HLA regions, among which 112 were reported to be associated with ATH in the NHGRI GWAS catalog (enriched, $p < 2.2e-16$). A pathway enrichment analysis revealed

12 significant pathways (Figure S8). Notably, a cluster of genes in 17q21 were identified by OWAS, including *ZPBP2, ORMDL3, GSDMB, GSDMA, IKZF3*, and *LRRC3C*. In this cluster, increased expression of *ORMDL3* and *GSDMB* leads to an increased airway hyperreactivity (AHR) [22], which is characteristic of ATH. *In vivo* studies have shown in *ZPBP2* knockout mice that a lack of *ZPBP2* protects against the development of HDM-induced airway inflammation and AHR [22].

In particular, OWAS identified three significant segments in risk locus 11q13.5, in the neighborhood of *LRRC32*. Although *LRRC32* has previously been associated with ATH [19], its biological mechanism is unclear. OWAS not only precisely identified the functional segment, but also associated the change of chromosome accessibility with the disease risk, suggesting that the association of the 11q13.5 risk locus might be attributed to epigenetic mechanisms. Interestingly, two of the three identified segments was in a distal enhancer (chr11: 76,297,475-76,311,165, highlighted in red in Figure S9), recently reported in Nasrallah et al. (2020). The distal enhancer affects susceptibility to autoimmune diseases by recruiting the transcription factors STAT5 and NF-κB to mediate signal-driven expression of *LRRC32*, which encodes the protein glycoprotein A repetitions predominant (GARP) [24]. This example underscores the capability of OWAS to interpret epigenetic mechanisms of noncoding variants in risk loci.

1.8 Comparison between OWAS segments and caQTLs

The caQTLs (ATAC-QTLs) in Gate et al. (2018) [13] and Kumasaka et al. (2016) [18] are genetic variants that affect the accessibility of the corresponding peak. The overlap of caQTLs (ATAC-QTLs) and GWAS loci can provide insights into how natural genetic variants modulate cis-regulatory elements, in isolation or in concert, to influence gene expression. However, as is discussed in the Introduction, experimental measurement of chromatin accessibility in large cohort is costly and therefore not common. Thus, the power to identify caQTLs is still limited. In fact, the number of caQTLs identified by Gate et al. is only 3,317 for local-ATAC-QTLs and 381 for distal-ATAC-QTLs, with 105 individuals in the cohort. Furthermore, to maximize statistical power, the analysis was restricted to 64,188 ATAC-peaks with at least one SNP. As a result, the genome coverage of current caQTL dataset is low.

One of the advantages of our method is to leverage the power of machine learning approaches for in silico predictions of chromatin accessibility from DNA sequences. OWAS can be applied at the genome scale. We take RA as an example to compare the two approaches. We found 1,343 GWAS SNPs ($p < 5e-08$) in OWAS segments, while only 8 in the caQTL dataset. Despite the limited power at current stage, we believe that caQTLs and related methodologies will be beneficial in bringing insights into disease etiologies, when more data become available.

As for the functional relevance, we performed pathway analysis on these common and exclusive SNPs of OWAS and caQTLs on RA analysis. We first overlapped the caQTLs with GWAS significant SNPs. We considered both genome-wide significance ($p < 5e-08$), and a more relaxed threshold ($p < 5e-02$), as there were too few GWAS signals detected by caQTL under a stringent threshold. The caQTL were mapped with the nearest genes by SNPsnap. We again focused on the eight pathways discussed in Section 3.4 that are directly or indirectly related with RA (Figure S15). We found that pathway enrichments of OWAS were not influenced by removing caQTL genes, while the caQTL genes are only enriched in Human papillomavirus infection pathway.

2 Supplementary Materials

2.1 GWAS datasets

The GWAS summary statistics we used are summarized in Table S2. The individual-level GWAS data of CD, HT, and RA were retrieved from the Wellcome Trust Case Control Consortium (WTCCC). For quality control, we removed variants with missing rate above 0.01, and individuals with missing rate greater than 0.1. In addition, we filtered out SNPs with minor allele frequency smaller than $5e-03$. SNPs that significantly deviate from Hardy-Weinberg equilibrium (HWE) with $p < 1e-05$ were also removed. For running DeepWAS, we imputed genotypes in WTCCC dataset. The SHAPEIT program [8] was used to obtain the phased haplotypes, and then MiniMac3 [7] was used to impute genotypes in the WTCCC dataset and Northwestern Nugene Project dataset. The 1000 Genomes Phase3 v5 [5] obtained from the University of Michigan Abecasis lab was used as the reference panel, with approximately 41 million imputed variants. We retained all variants with an imputation R^2>0.8 [12], yielding about 7 million variants.

2.2 Chromatin accessibility data

We verified that differences of chromosome accessibility between genomic segments can be captured by *in silico* predictions using publicly available data for three histone modifications including H3K4me1, H3K4me3, and H3K27ac, in HapMap lymphoblastoid cell lines (LCLs) of the HapMap Yoruba (YRI) collection from the ENCODE project [21, 11].

2.3 TWAS results

We downloaded the FUSION software [14] along with its prepackaged weights for gene expression data to compute TWAS results of Young Finns Study blood cell line, using the same GWAS summary statistics with OWAS (described in Table S2).

3 Supplementary Tables

Table S1: **Summary information of the 12 common human cell types from UW ENCODE and the corresponding tissues in OWAS analysis.**

Cell type	Tissue	Description
A549	Epithelium	Human lung carcinoma derived epithelial cell line
GM12878	Blood	Lymphoblastoid
HeLa-S3	Cervix	Adherent human cervical adenocarcinoma
HepG2	Liver	Hepatocellular carcinoma
HMEC	Breast	Mammary epithelial
HSMM	Muscle	Human skeletal muscle myoblasts
HUVEC	Blood vessel	Umbilical vein endothelial
K562	Blood	Myeloid
LNCaP	Prostate	Prostate adenocarcinoma
MCF-7	Breast	Mammary gland adenocarcinoma
NHEK	Skin	Normal epidermal keratinocytes
Th1	Blood	T1 helper

Table S2: **GWAS summary statistics and cell types used in OWAS analysis.** The relevant cell types were selected with GARFIELD.

Trait	Data	N_{case}	$N_{control}$	Cell type
Asthma (ATH)	UK Biobank	41,934	319,207	Th1
Crohn's disease (CD)	IBDG	20,550	41,642	Th1
High cholesterol (HC)	UK Biobank	43,957	317,184	Th1
Hypertension (HT)	UK Biobank	93,560	267,581	Huvec
Prostate cancer (PrCa)	Schumacher et al. (2018)	46,939	27,910	Huvec
Rheumatoid arthritis (RA)	Okada et al. (2014)	14,361	43,923	GM12878

Table S3: **Jaccard index and Spearman's rank correlation between significant segments/genes identified with GM12878 and other 11 cell types on RA analysis.**

	Segment		Gene	
	Jaccard index	Spearman's correlation	Jaccard index	Spearman's correlation
A549	0.467	0.350	0.700	0.834
HeLa-S3	0.474	0.338	0.721	0.799
HepG2	0.444	0.316	0.694	0.831
HMEC	0.472	0.357	0.703	0.822
HSMM	0.473	0.359	0.674	0.820
HUVEC	0.487	0.386	0.702	0.822
K562	0.456	0.331	0.713	0.830
LNCaP	0.459	0.355	0.711	0.817
MCF-7	0.455	0.328	0.672	0.792
NHEK	0.470	0.365	0.658	0.784
Th1	0.523	0.478	0.687	0.823

Table S4: **Top 10 significant OWAS genes for RA.** p-GWAS lists p-values documented in GWAS catalog. The enhancer information is matched by EAGLE. Literature lists the discussion of the association between the identified gene and RA.

Region	Gene	Z score	p-OWAS	p-GWAS[1]	Enhancer	Literature
12q13.3	*INHBC*	-306.21	$<5.0e-100$	NA	No	[30]
7q11.23	*LOC101926943*	44.29	$<5.0e-100$	NA	No	[23]
1p13.2	*PHTF1*	-23.44	$<5.0e-100$	$6.0e-25$	Yes	[6, 28]
18q11.2	*LAMA3*	18.36	$3.0e-75$	NA	No	[3]
1p13.2	*HIPK1*	17.29	$5.6e-67$	$5.0e-8$	No	[9]
1p13.2	*PTPN22*	16.93	$2.7e-64$	$<5.0e-100$	Yes	[27]
1p13.2	*AP4B1*	16.82	$1.8e-63$	NA	Yes	[1]
1p13.2	*RSBN1*	-16.75	$5.7e-63$	$6.0e-25$	Yes	[6, 28]
1p13.2	*BCL2L15*	-16.52	$2.8e-61$	NA	Yes	[31]
1p13.2	*OLFML3*	-16.52	$2.8e-61$	NA	No	[20]

[1] NA means the gene is not reported to be significantly associated with RA in GWAS catalog.

Table S5: **Mean and standard error (in brackets) of type I error rate for 1,000 simulations under different simulation settings.** α is the p-value threshold. l_s is the number of SNPs in a segment.

l_s	$\alpha = 0.01$	$\alpha = 0.05$	$\alpha = 0.10$
5	$9.87e-03$ ($3.12e-03$)	$5.03e-02$ ($6.54e-03$)	$9.97e-02$ ($9.50e-03$)
20	$9.88e-03$ ($3.08e-03$)	$5.02e-02$ ($6.33e-03$)	$1.00e-01$ ($9.37e-03$)
50	$1.04e-02$ ($3.57e-03$)	$5.04e-02$ ($7.02e-03$)	$1.00e-01$ ($9.60e-03$)

Table S6: **The number of significant and non-significant segments (genome wide significance) within and outside the 100 KB up and down-stream from the transcription start sites (TSS).**

	# Significant segments	# Non-significant segments
100 KB up and down-stream from the TSS	682	330,053
Outside of 100KB up and down-stream from the TSS	71	204,485

4 Supplementary Figures

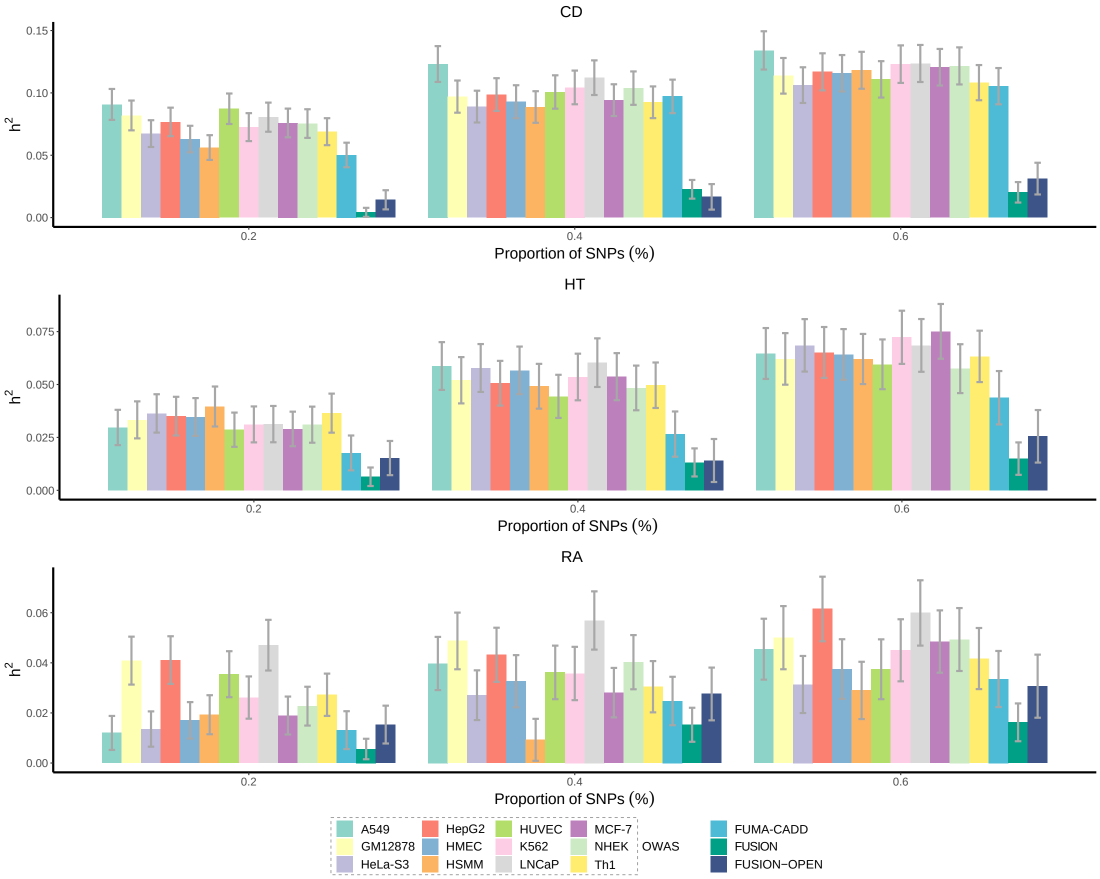

Figure S1: **Enrichments for heritability with varying segment lengths in OWAS analysis.** Other three methods including FUSION genes, FUSION genes in open regions, and SNPs prioritized by FUMA-CADD scores. OWAS segments and FUSION genes were ranked by their *p*-values, and the proportion of SNPs (x-axis) and the explained heritability (y-axis) at varying cutoffs are displayed. The error bars correspond to the standard error of the heritability estimated by GCTA software. The discovery cohorts are derived using UKB summary statistics and the heritability was estimated with the WTCCC individual-level genotype data.

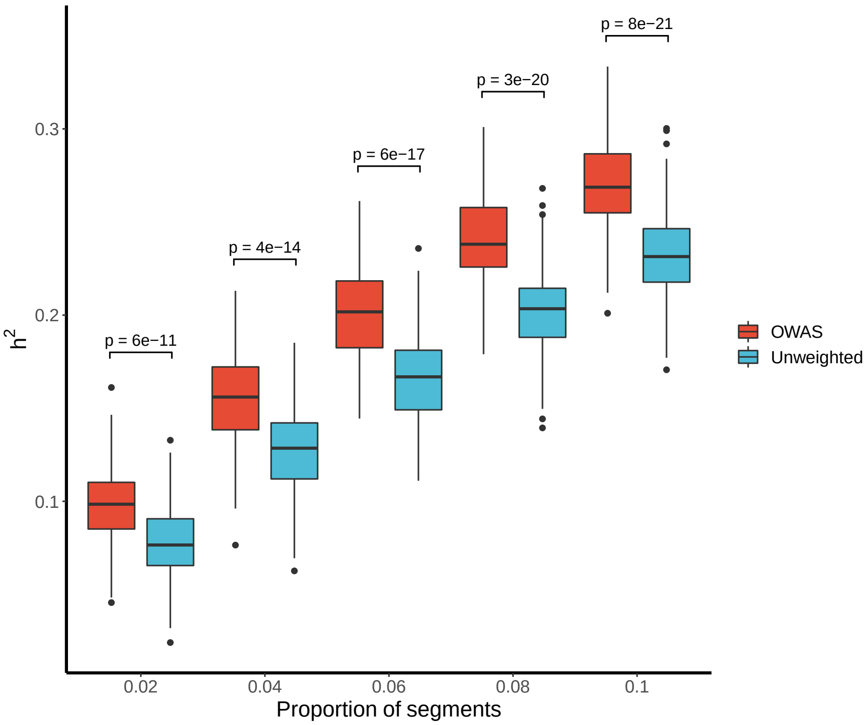

Figure S2: **OWAS segments explain more heritability in simulations.** In the unweighted model, weights of all SNPs were set to be equal. The simulations were run for 100 times. The p-value indicates the significance level for the t test between means of heritability in OWAS segments and unweighted segments.

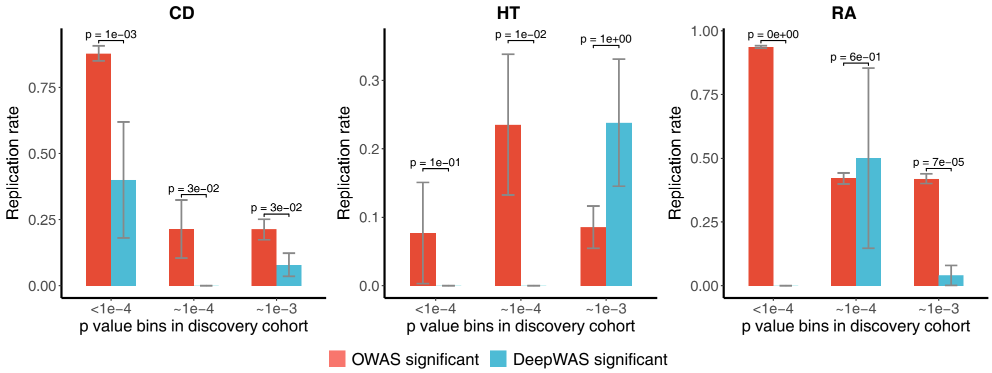

Figure S3: **Replication rate of OWAS and DeepWAS results.** Both methods were trained with imputed WTCCC data, and the UKB data were used as the replication cohorts. In the discovery cohort, GWAS were divided into 3 bins according to their p-values. In the replication cohort, GWAS significant SNPs were identified with a relaxed threshold ($p < 5e - 02$). In each bin, the SNPs were broken down into prioritized groups by the OWAS and DeepWAS results.

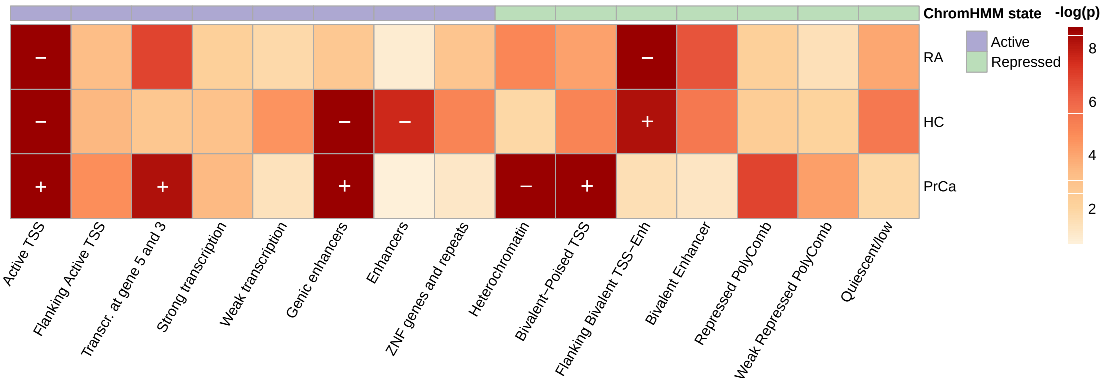

Figure S4: **Enrichment of OWAS segments for RA, HC, and PrCa with 15 chromHMM states.** The heatmap shows the -log p-values for each pair of trait and chromatin state. The “+” and “-” indicate the significant enriched/depleted results, which were derived from the permutation test address the potential prioritization bias towards SNPs that overlap with DNase-seq peaks. The predicted openness effects of the SNPs within and outside the DNase-seq peaks were randomized, separately, for 5,000 times.

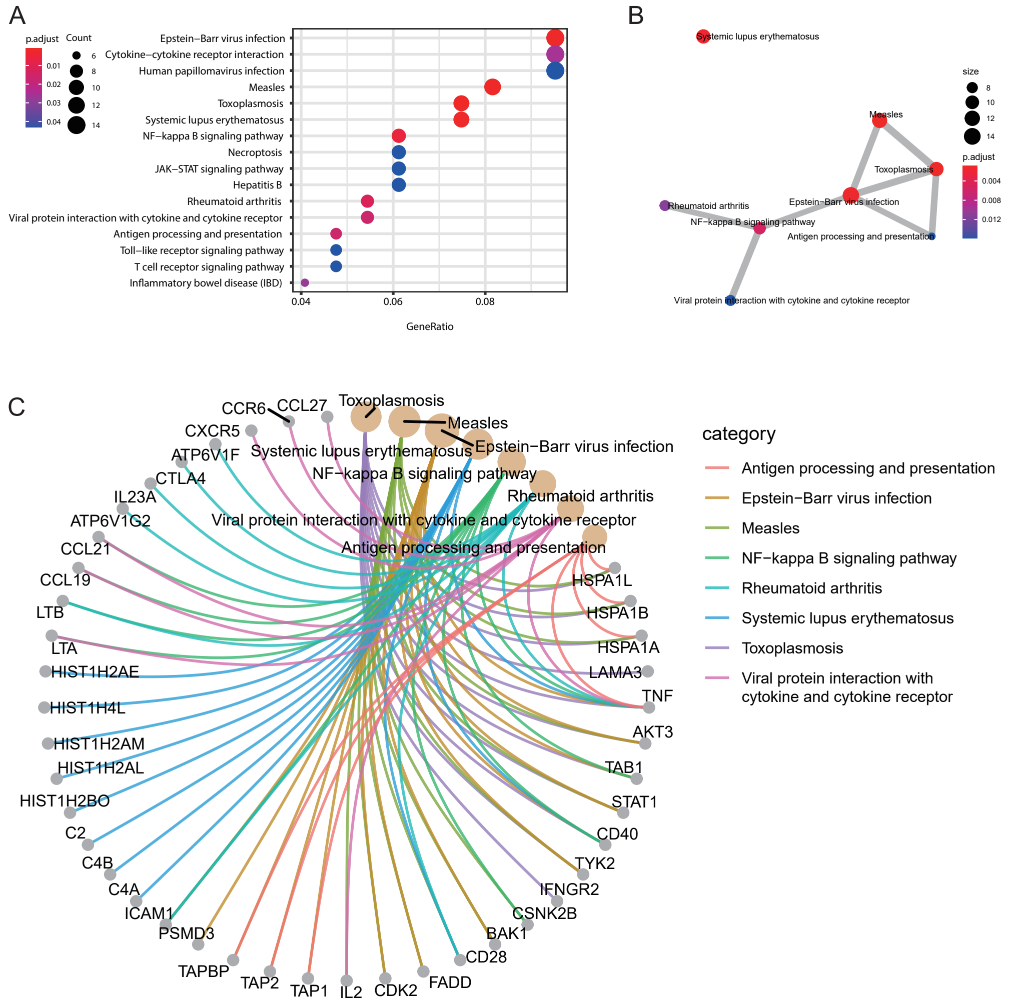

Figure S5: **KEGG pathway enrichment analysis results of 397 RA associated genes identified by OWAS ($p < 5e-08$).** (A) Dot plot of the enrichment analysis. The enrichment scores and gene ratio are depicted as the color and the horizon distance between the dots and y axis. The gene counts were encoded as dot sizes. (B) Enrichment map of the enrichment analysis. The enriched pathways were linked if there were overlapping gene sets. A functional module containing seven pathways was shown in the figure. (C) Network plot of identified genes and the enriched pathways. The pathways are displayed in different colors and genes are linked with the enriched pathways with corresponding colors.

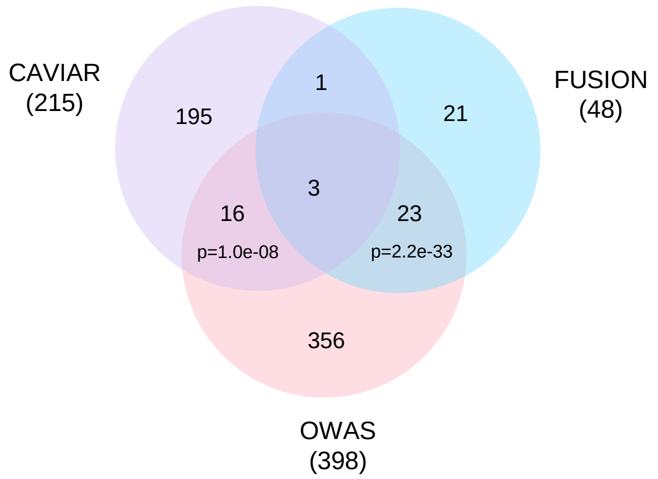

Figure S6: **Venn diagram of significant genes identified by OWAS, CAVIAR, and FUSION in RA analysis.** CAVIAR and FUSION genes were significantly enriched in OWAS genes.

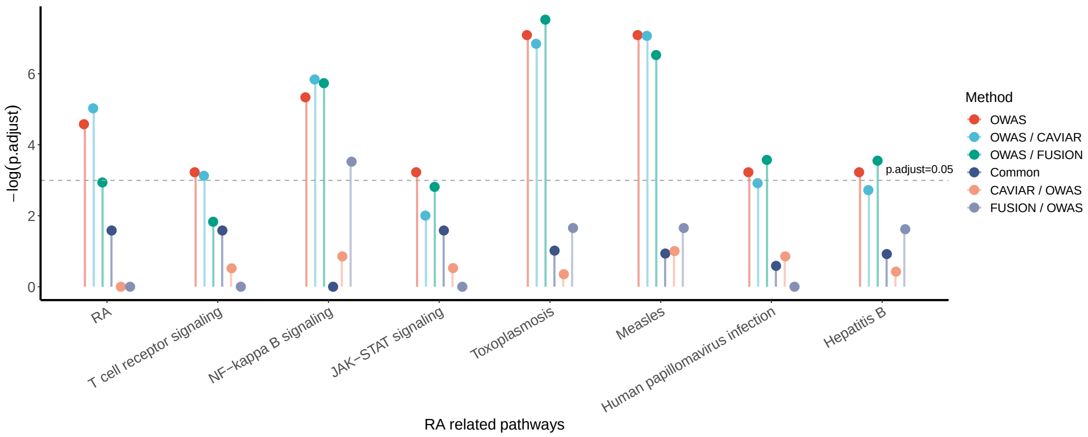

Figure S7: **Pathway enrichments of OWAS genes, OWAS genes with CAVIAR genes removed, OWAS genes with TWAS genes removed, common genes, CAVIAR and TWAS genes with OWAS genes removed in RA analysis.** The common genes are defined by genes identified by no less than two methods. Eight RA related pathways discussed in Section 3.4 are compared.

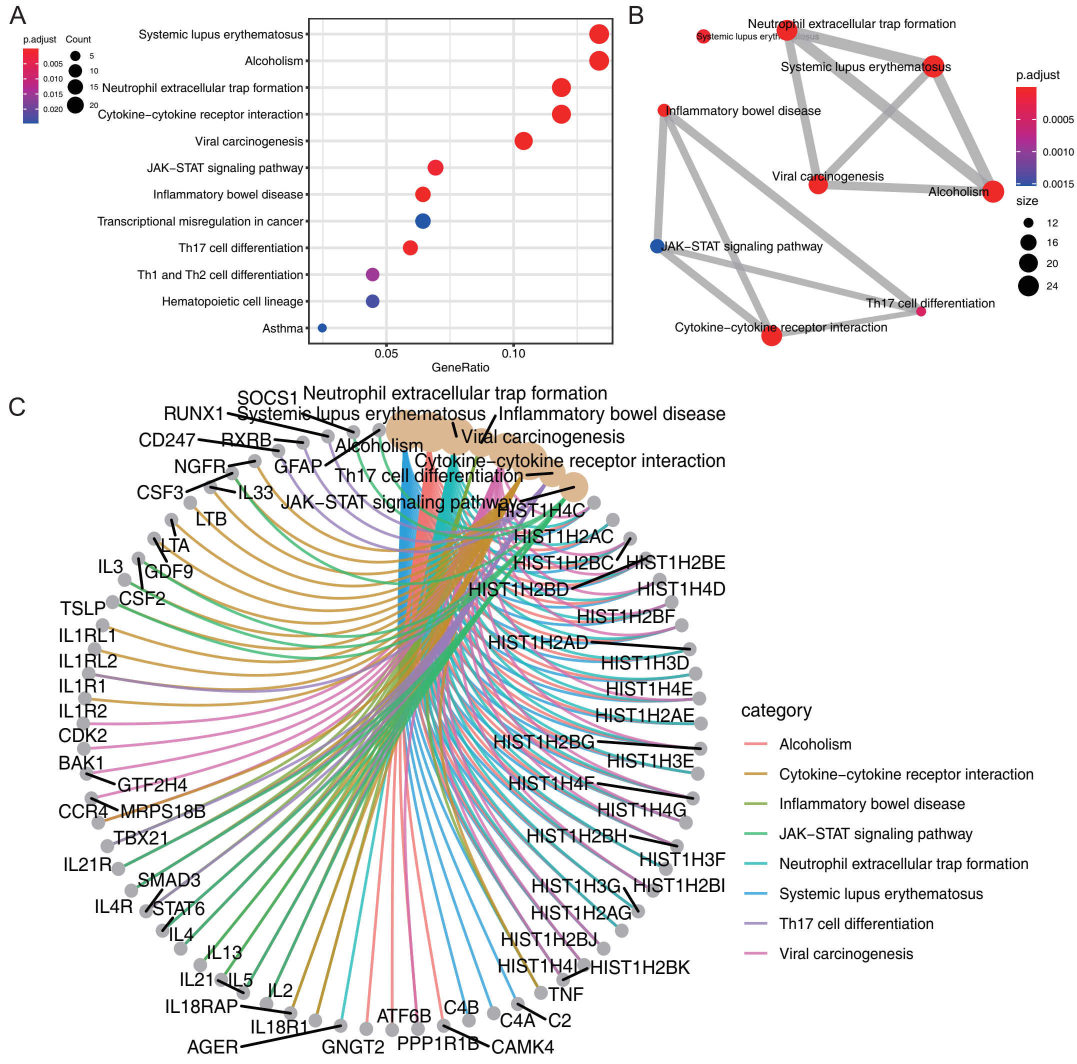

Figure S8: **KEGG pathway enrichment analysis results of 516 ATH associated genes identified by OWAS ($p < 5e-08$).** (A) Dot plot of the enrichment analysis. The enrichment scores and gene ratio are depicted as the color and the horizon distance between the dots and y axis. The gene counts were encoded as dot sizes. (B) Enrichment map of the enrichment analysis. The enriched pathways were linked if there were overlapping gene sets. A functional module containing seven pathways was shown in the figure. (C) Network plot of identified genes and the enriched pathways. The pathways are displayed in different colors and genes are linked with the enriched pathways with corresponding colors.

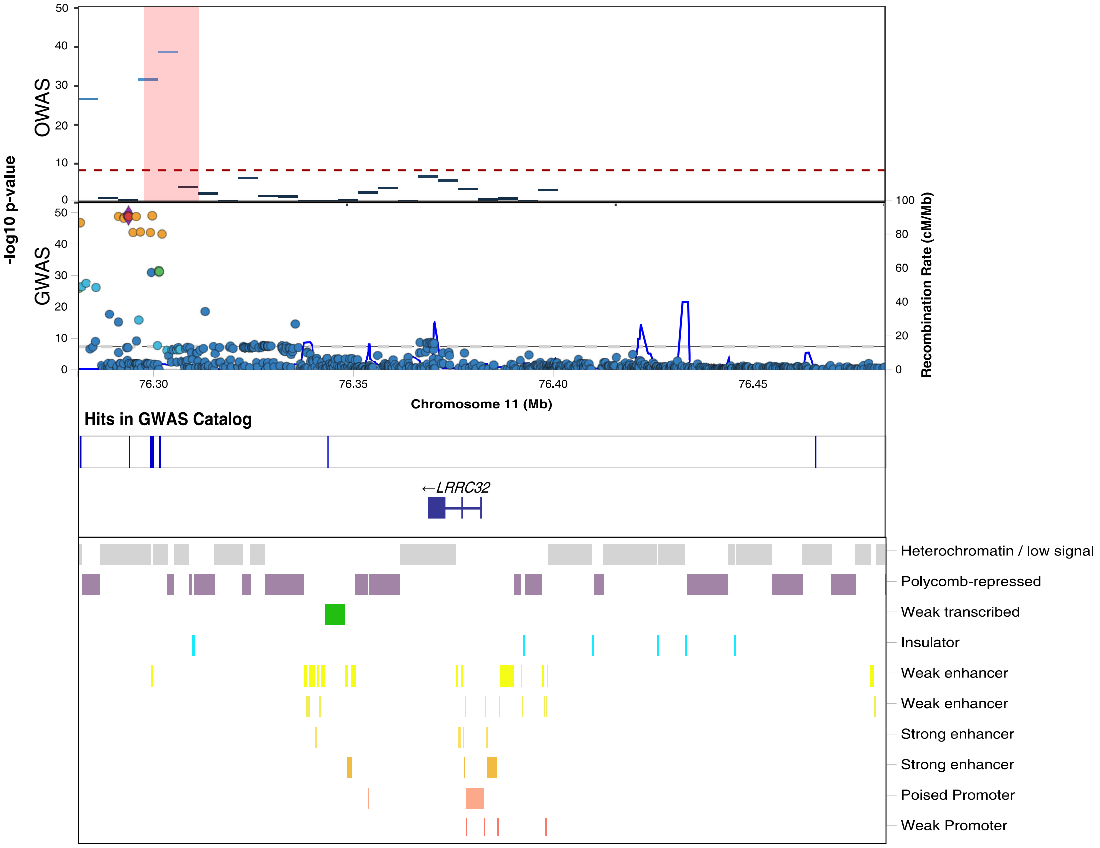

Figure S9: **Locuszoom in 100 KB up and down-stream from the TSS of *LRRC32*.** The two Manhattan plots at the top panel show the association p values of OWAS and GWAS results in the 11q13.5 locus. The location of the distal enhancer is highlighted in red. Hits in GWAS catalog, gene regions and chromatin states are annotated in the bottom of the figure. OWAS identified three significant segments among the regulatory region of *LRRC32*, two of which is overlapped with the newly reported risk locus of ATH.

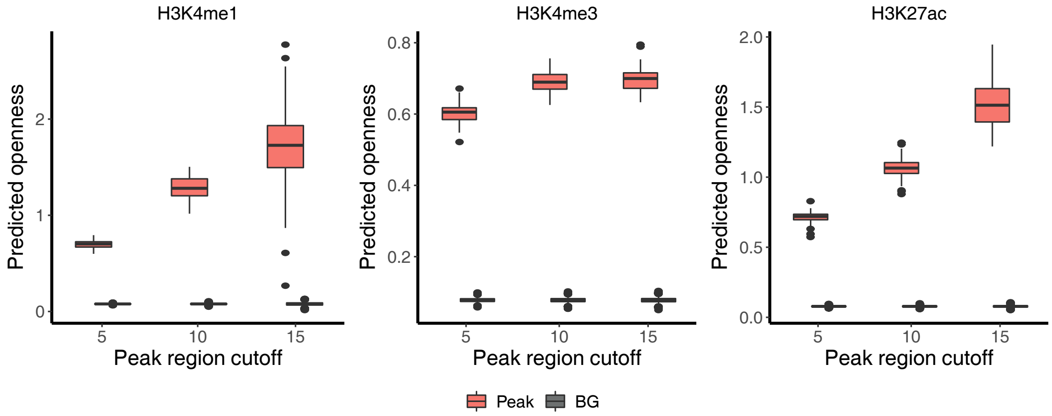

Figure S10: **Predicted openness in active chromatin region.** Different thresholds were set for the experimental peak signals. The average of predicted openness for variants locating in the selected peaks, and those randomly sampled were compared. The sampling process was repeated for 10,000 times. The averages of predicted openness increased with more stringent thresholds chosen. Through all three histone modifications, the predicted openness in peak regions differed significantly from the background.

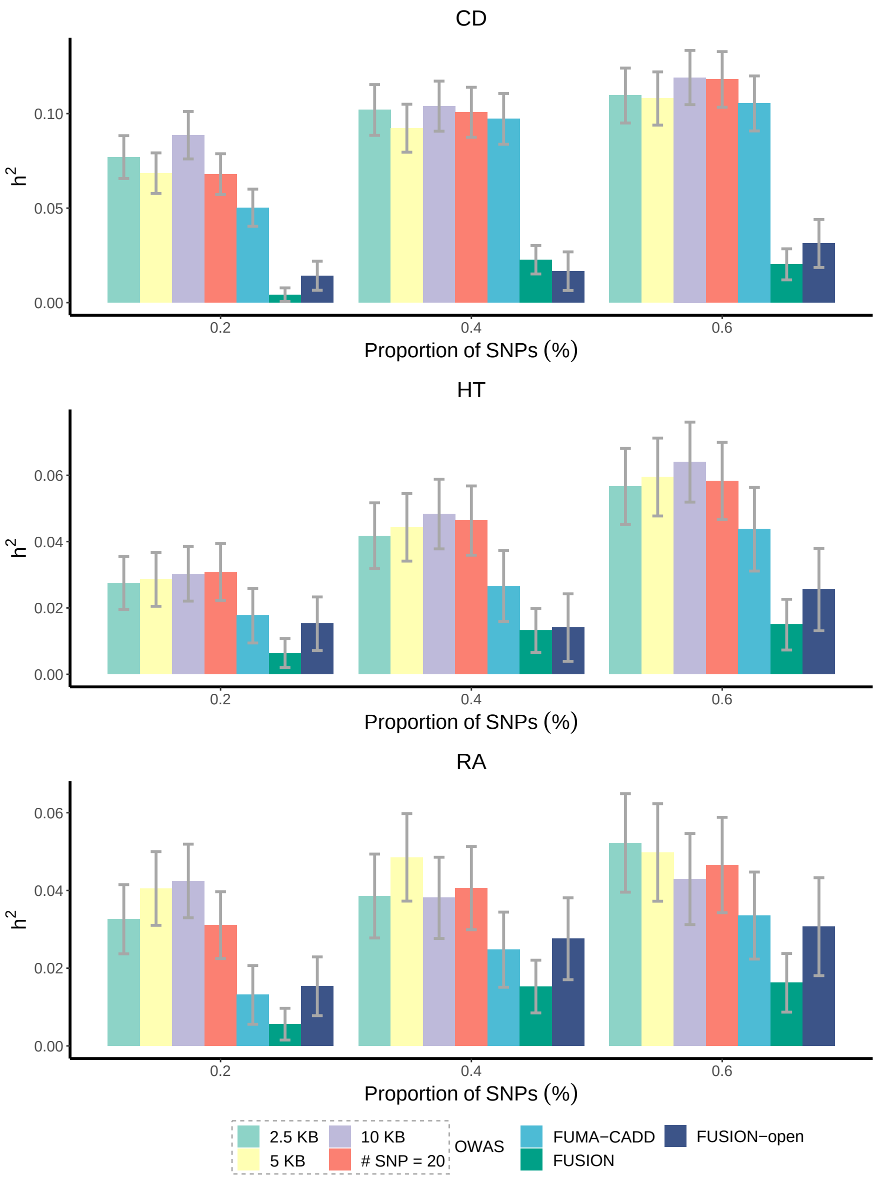

Figure S11: **Enrichments for heritability with varying segment lengths in OWAS analysis. Other three methods including FUSION genes, FUSION genes in open regions, and SNPs prioritized by FUMA-CADD scores.** OWAS segments and FUSION genes were ranked by their p-values, and the proportion of SNPs (x-axis) and the explained heritability (y-axis) at varying cutoffs are displayed. The error bars correspond to the standard error of the heritability estimated by GCTA software. The discovery cohorts are derived using UKB summary statistics and the heritability was estimated with the WTCCC individual-level genotype data.

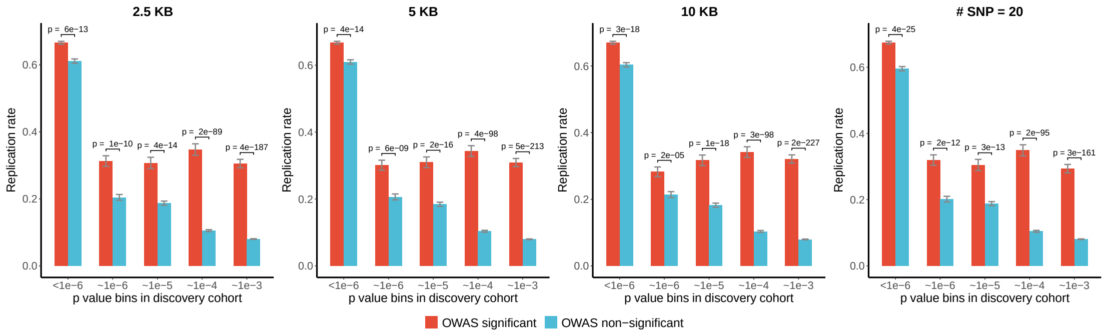

Figure S12: **Replication rate of OWAS results with varying segment lengths on RA analysis.** OWAS were performed with GWAS summary statistics from both discovery cohort (with larger sample sizes) and the replication cohort in RA. In the discovery cohort, GWAS were divided into 5 bins according to their p-values. In the replication cohort, GWAS significant SNPs were identified with a relaxed threshold ($p < 5e-02$). In each bin, the SNPs were broken down into prioritized and not prioritized groups by the OWAS results.

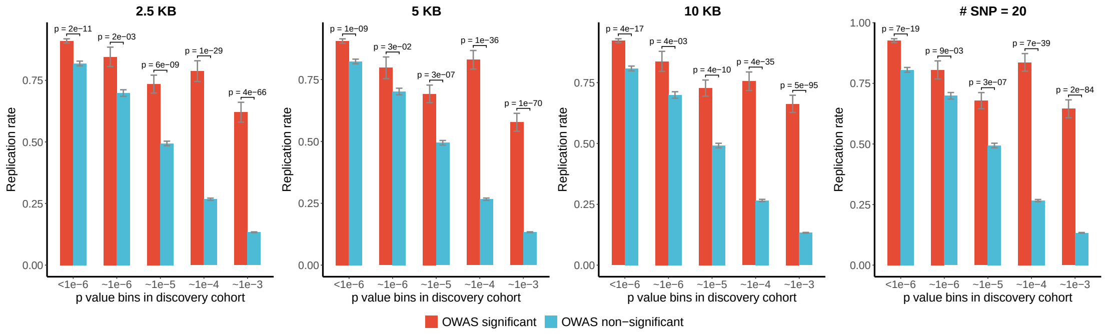

Figure S13: **Replication rate of OWAS results with varying segment lengths on HC analysis.** OWAS were performed with GWAS summary statistics from both discovery cohort (with larger sample sizes) and the replication cohort in HC. In the discovery cohort, GWAS were divided into 5 bins according to their p-values. In the replication cohort, GWAS significant SNPs were identified with a relaxed threshold ($p < 5e-02$). In each bin, the SNPs were broken down into prioritized and not prioritized groups by the OWAS results.

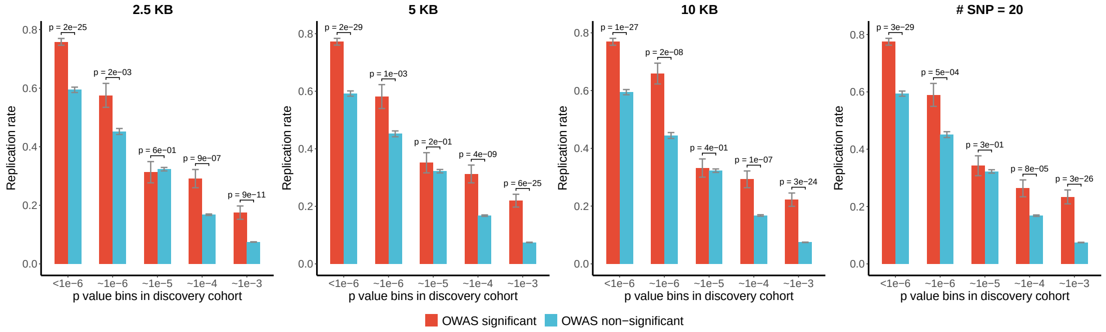

Figure S14: **Replication rate of OWAS results with varying segment lengths on PrCa analysis.** OWAS were performed with GWAS summary statistics from both discovery cohort (with larger sample sizes) and the replication cohort in PrCa. In the discovery cohort, GWAS were divided into 5 bins according to their p-values. In the replication cohort, GWAS significant SNPs were identified with a relaxed threshold ($p < 5e-02$). In each bin, the SNPs were broken down into prioritized and not prioritized groups by the OWAS results.

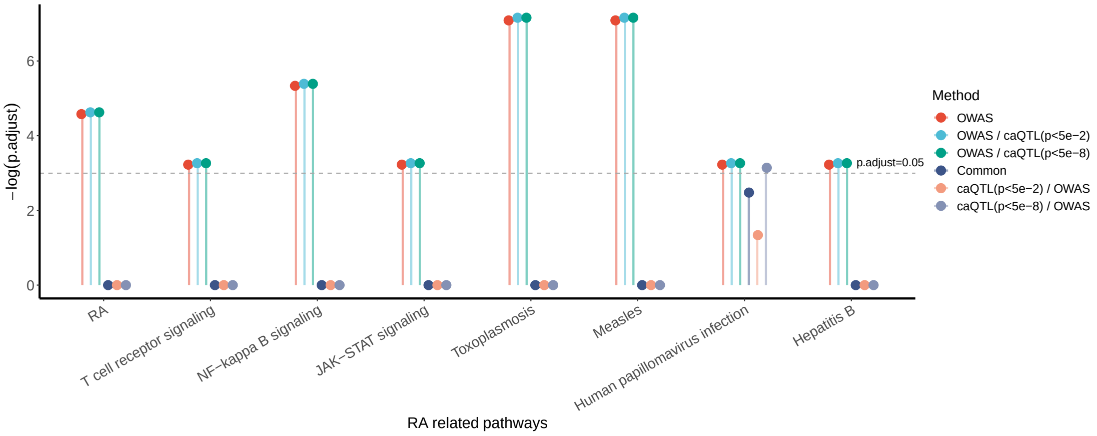

Figure S15: **Enrichment analysis of RA related pathways for OWAS genes, OWAS genes with caQTL genes removed, common genes, and caQTL genes with OWAS gene removed in RA analysis.** The caQTLs were mapped to the nearest genes with SNPsnap. Two thresholds were considered as caQTL detected few signals under stringent GWAS cutoffs. Common genes are defined by genes identified by no less than two methods.

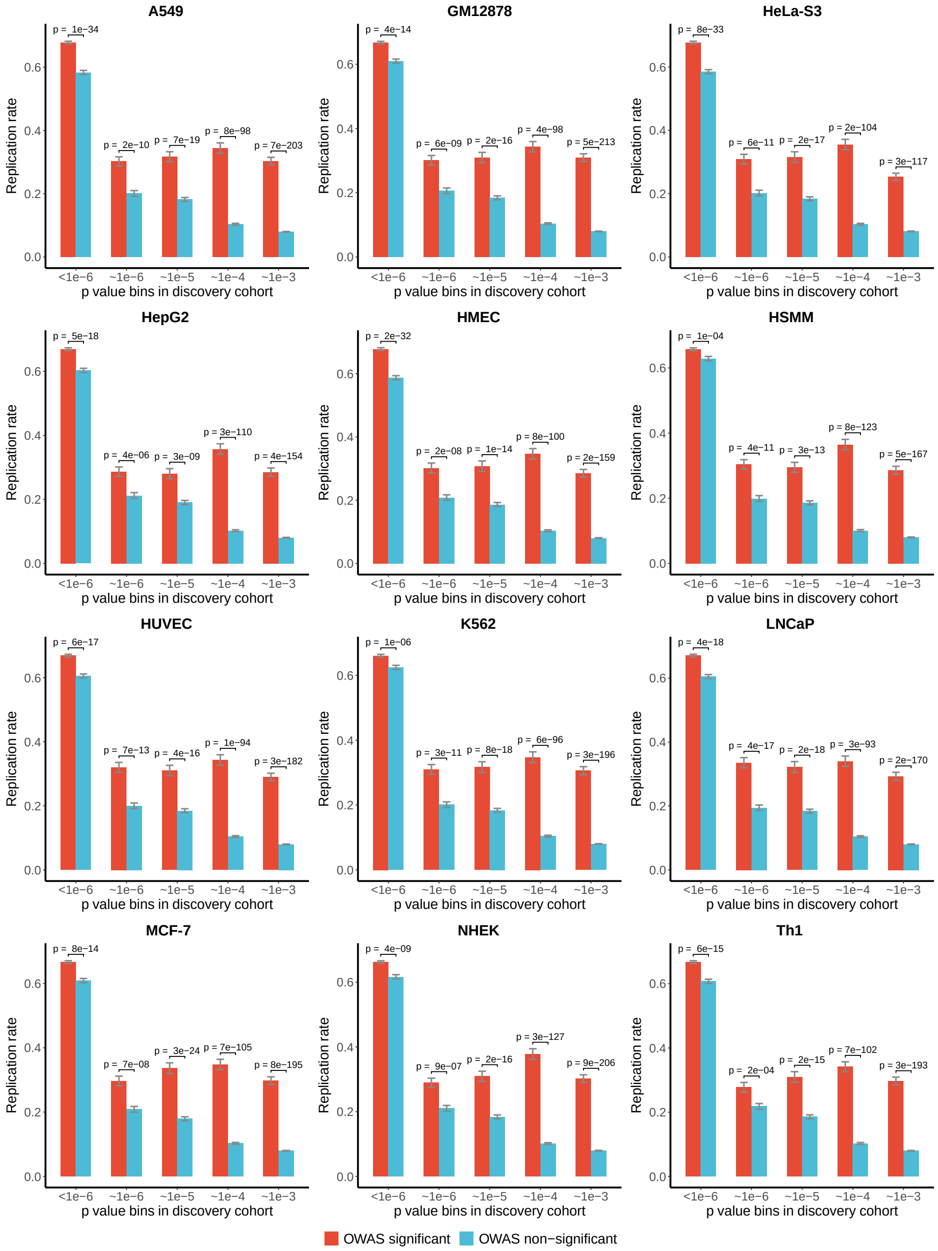

Figure S16: **Replication rate of OWAS results with 12 cell types from different tissues on RA analysis.** OWAS was performed with GWAS summary statistics from both discovery cohort (with larger sample sizes) and the replication cohort in RA. In the discovery cohort, GWAS SNPs were divided into 5 bins according to their p-values. In the replication cohort, GWAS significant SNPs were identified with a relaxed threshold ($p < 5e-02$). In each bin, the SNPs were broken down into prioritized and not prioritized groups by the OWAS results.

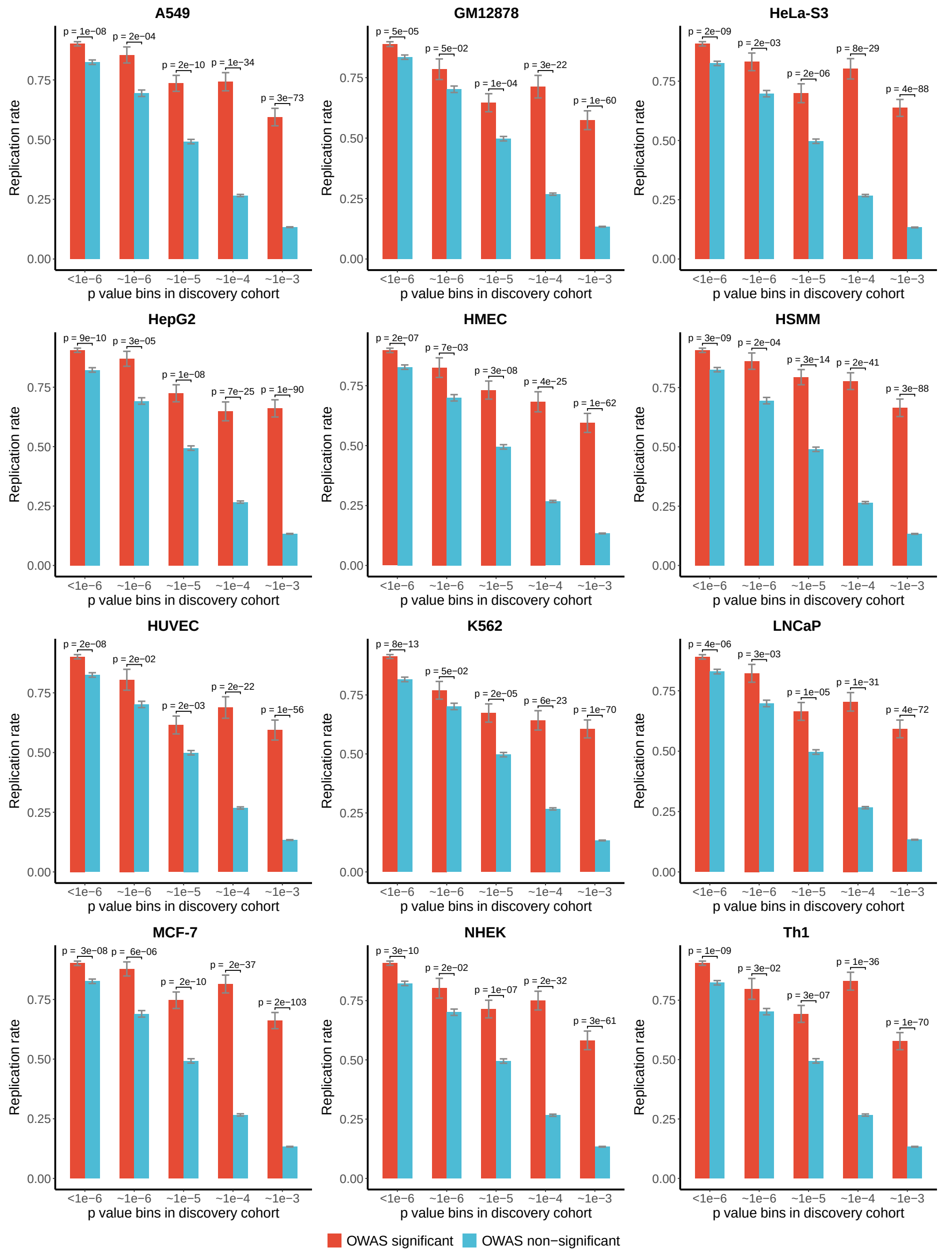

Figure S17: **Replication rate of OWAS results with 12 cell types from different tissues on HC analysis.** OWAS was performed with GWAS summary statistics from both discovery cohort (with larger sample sizes) and the replication cohort in HC. In the discovery cohort, GWAS SNPs were divided into 5 bins according to their p-values. In the replication cohort, GWAS significant SNPs were identified with a relaxed threshold ($p < 5e - 02$). In each bin, the SNPs were broken down into prioritized and not prioritized groups by the OWAS results.

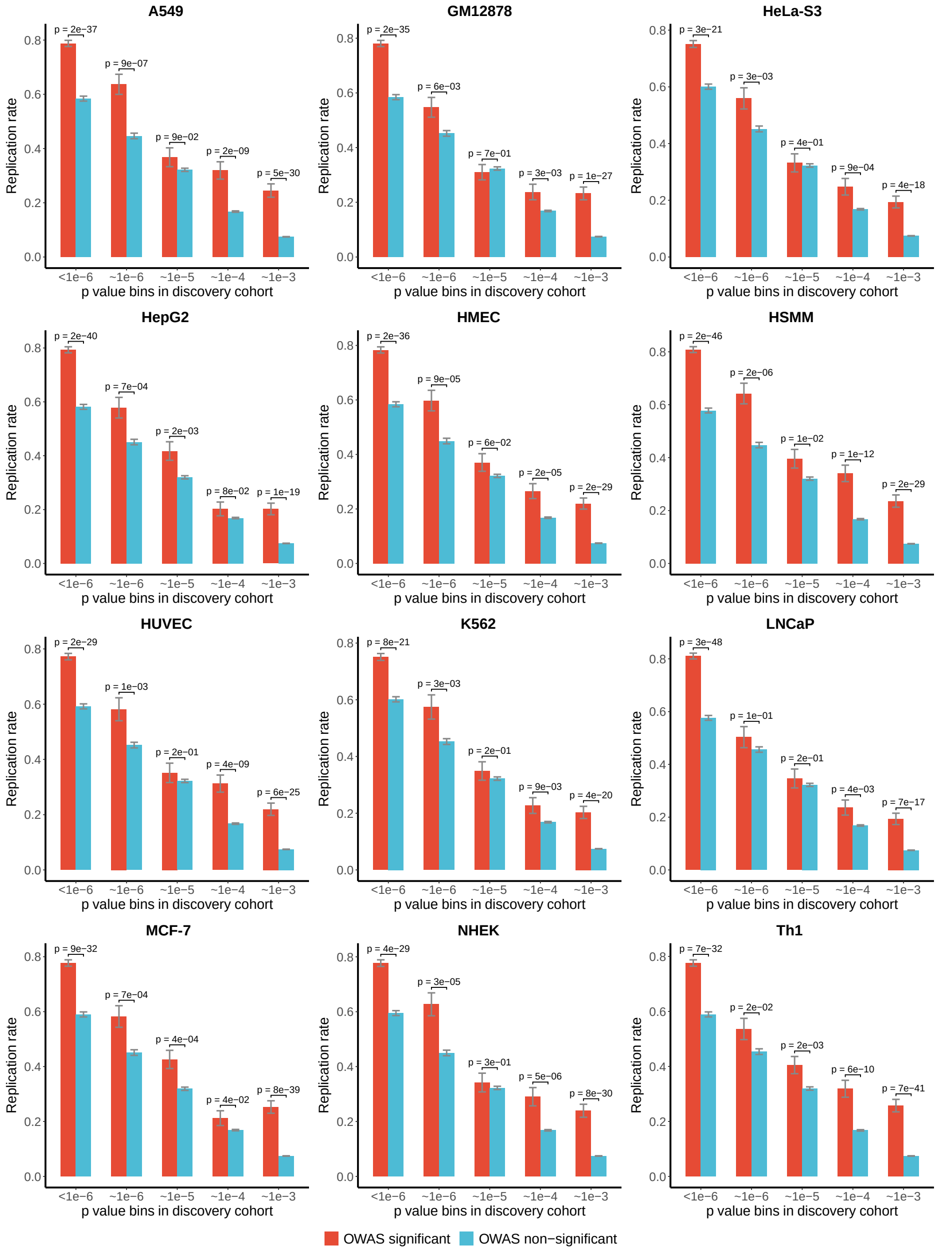

Figure S18: **Replication rate of OWAS results with 12 cell types from different tissues on PrCa analysis.** OWAS was performed with GWAS summary statistics from both discovery cohort (with larger sample sizes) and the replication cohort in PrCa. In the discovery cohort, GWAS SNPs were divided into 5 bins according to their p-values. In the replication cohort, GWAS significant SNPs were identified with a relaxed threshold ($p < 5e - 02$). In each bin, the SNPs were broken down into prioritized and not prioritized groups by the OWAS results.

References

[1] Marialbert Acosta-Herrera, Martin Kerick, David González-Serna, Cisca Wijmenga, Andre Franke, Peter K Gregersen, Leonid Padyukov, Jane Worthington, Timothy James Vyse, Marta Eugenia Alarcón-Riquelme, et al. Genome-wide meta-analysis reveals shared new loci in systemic seropositive rheumatic diseases. *Annals of the Rheumatic Diseases*, 78(3):311–319, 2019.

[2] Janine Arloth, Gökcen Eraslan, Till FM Andlauer, Jade Martins, Stella Iurato, Brigitte Kühnel, Melanie Waldenberger, Josef Frank, Ralf Gold, Bernhard Hemmer, et al. DeepWAS: Multivariate genotype-phenotype associations by directly integrating regulatory information using deep learning. *PLoS Computational Biology*, 16(2):e1007616, 2020.

[3] William J Astle, Heather Elding, Tao Jiang, Dave Allen, Dace Ruklisa, Alice L Mann, Daniel Mead, Heleen Bouman, Fernando Riveros-Mckay, Myrto A Kostadima, et al. The allelic landscape of human blood cell trait variation and links to common complex disease. *Cell*, 167(5):1415–1429, 2016.

[4] Alvaro N Barbeira, Scott P Dickinson, Rodrigo Bonazzola, Jiamao Zheng, Heather E Wheeler, Jason M Torres, Eric S Torstenson, Kaanan P Shah, Tzintzuni Garcia, Todd L Edwards, et al. Exploring the phenotypic consequences of tissue specific gene expression variation inferred from GWAS summary statistics. *Nature Communications*, 9(1):1825, 2018.

[5] 1000 Genomes Project Consortium et al. A global reference for human genetic variation. *Nature*, 526(7571):68, 2015.

[6] Wellcome Trust Case Control Consortium et al. Genome-wide association study of 14,000 cases of seven common diseases and 3,000 shared controls. *Nature*, 447(7145):661, 2007.

[7] Sayantan Das, Lukas Forer, Sebastian Schönherr, Carlo Sidore, Adam E Locke, Alan Kwong, Scott I Vrieze, Emily Y Chew, Shawn Levy, Matt McGue, et al. Next-generation genotype imputation service and methods. *Nature Genetics*, 48(10):1284–1287, 2016.

[8] Olivier Delaneau, Jonathan Marchini, and Jean-François Zagury. A linear complexity phasing method for thousands of genomes. *Nature Methods*, 9(2):179–181, 2012.

[9] David Ellinghaus, Luke Jostins, Sarah L Spain, Adrian Cortes, Jörn Bethune, Buhm Han, Yu Rang Park, Soumya Raychaudhuri, Jennie G Pouget, Matthias Hübenthal, et al. Analysis of five chronic inflammatory diseases identifies 27 new associations and highlights disease-specific patterns at shared loci. *Nature Genetics*, 48(5):510, 2016.

[10] Jason Ernst and Manolis Kellis. Chromatin-state discovery and genome annotation with chromHMM. *Nature Protocols*, 12(12):2478, 2017.

[11] Jason Ernst, Pouya Kheradpour, Tarjei S Mikkelsen, Noam Shoresh, Lucas D Ward, Charles B Epstein, Xiaolan Zhang, Li Wang, Robbyn Issner, Michael Coyne, et al. Mapping and analysis of chromatin state dynamics in nine human cell types. *Nature*, 473(7345):43, 2011.

[12] Eric R Gamazon, Heather E Wheeler, Kaanan P Shah, Sahar V Mozaffari, Keston Aquino-Michaels, Robert J Carroll, Anne E Eyler, Joshua C Denny, Dan L Nicolae, Nancy J Cox, et al. A gene-based association method for mapping traits using reference transcriptome data. *Nature Genetics*, 47(9):1091, 2015.

[13] Rachel E Gate, Christine S Cheng, Aviva P Aiden, Atsede Siba, Marcin Tabaka, Dmytro Lituiev, Ido Machol, M Grace Gordon, Meena Subramaniam, Muhammad Shamim, et al. Genetic determinants of co-accessible chromatin regions in activated T cells across humans. *Nature Genetics*, 50(8):1140–1150, 2018.

[14] Alexander Gusev, Arthur Ko, Huwenbo Shi, Gaurav Bhatia, Wonil Chung, Brenda WJH Penninx, Rick Jansen, Eco JC De Geus, Dorret I Boomsma, Fred A Wright, et al. Integrative approaches for large-scale transcriptome-wide association studies. *Nature Genetics*, 48(3):245, 2016.

[15] Alexander Gusev, Nicholas Mancuso, Hyejung Won, Maria Kousi, Hilary K Finucane, Yakir Reshef, Lingyun Song, Alexias Safi, Steven McCarroll, Benjamin M Neale, et al. Transcriptome-wide association study of schizophrenia and chromatin activity yields mechanistic disease insights. *Nature Genetics*, 50(4):538, 2018.

[16] Farhad Hormozdiari, Emrah Kostem, Eun Yong Kang, Bogdan Pasaniuc, and Eleazar Eskin. Identifying causal variants at loci with multiple signals of association. *Genetics*, 198(2):497–508, 2014.

[17] Valentina Iotchkova, Graham RS Ritchie, Matthias Geihs, Sandro Morganella, Josine L Min, Klaudia Walter, Nicholas John Timpson, Ian Dunham, Ewan Birney, and Nicole Soranzo. GARFIELD classifies disease-relevant genomic features through integration of functional annotations with association signals. *Nature Genetics*, 51(2):343–353, 2019.

[18] Natsuhiko Kumasaka, Andrew J Knights, and Daniel J Gaffney. Fine-mapping cellular QTLs with RASQUAL and ATAC-seq. *Nature Genetics*, 48(2):206–213, 2016.

[19] Xingnan Li, Elizabeth J Ampleford, Timothy D Howard, Wendy C Moore, Huashi Li, William W Busse, Mario Castro, Serpil C Erzurum, Anne M Fitzpatrick, Benjamin Gaston, et al. The C11orf30-LRRC32 region is associated with total serum IgE levels in asthmatic patients. *Journal of Allergy and Clinical Immunology*, 129(2):575–578, 2012.

[20] Hongchao Lv, Mingming Zhang, Zhenwei Shang, Jin Li, Shanshan Zhang, Duan Lian, and Ruijie Zhang. Genome-wide haplotype association study identify the FGFR2 gene as a risk gene for acute myeloid leukemia. *Oncotarget*, 8(5):7891, 2017.

[21] Ryan McDaniell, Bum-Kyu Lee, Lingyun Song, Zheng Liu, Alan P Boyle, Michael R Erdos, Laura J Scott, Mario A Morken, Katerina S Kucera, Anna Battenhouse, et al. Heritable individual-specific and allele-specific chromatin signatures in humans. *Science*, 328(5975):235–239, 2010.

[22] Marina Miller, Christine Vuong, Meghan Farrell Garcia, Peter Rosenthal, Sudipta Das, Ning Weng, Alexa Pham, Yu Jin Kim, and David H Broide. Does reduced zona pellucida binding protein 2 (ZPBP2) expression on chromosome 17q21 protect against asthma? *Journal of Allergy and Clinical Immunology*, 142(2):706–709, 2018.

[23] David L Morris, Yujun Sheng, Yan Zhang, Yong-Fei Wang, Zhengwei Zhu, Philip Tombleson, Lingyan Chen, Deborah S Cunninghame Graham, James Bentham, Amy L Roberts, et al. Genome-wide association meta-analysis in Chinese and European individuals identifies ten new loci associated with systemic lupus erythematosus. *Nature Genetics*, 48(8):940, 2016.

[24] Rabab Nasrallah, Charlotte J Imianowski, Lara Bossini-Castillo, Francis M Grant, Mikail Dogan, Lindsey Placek, Lina Kozhaya, Paula Kuo, Firas Sadiyah, Sarah K Whiteside, et al. A distal enhancer at risk locus 11q13. 5 promotes suppression of colitis by T reg cells. *Nature*, pages 1–6, 2020.

[25] Joseph Nasser, Drew T Bergman, Charles P Fulco, Philine Guckelberger, Benjamin R Doughty, Tejal A Patwardhan, Thouis R Jones, Tung H Nguyen, Jacob C Ulirsch, Fritz Lekschas, et al. Genome-wide enhancer maps link risk variants to disease genes. *Nature*, 593(7858):238–243, 2021.

[26] Tune H Pers, Pascal Timshel, and Joel N Hirschhorn. SNPsnap: a Web-based tool for identification and annotation of matched SNPs. *Bioinformatics*, 31(3):418–420, 2015.

[27] Robert M Plenge, Mark Seielstad, Leonid Padyukov, Annette T Lee, Elaine F Remmers, Bo Ding, Anthony Liew, Houman Khalili, Alamelu Chandrasekaran, Leela RL Davies, et al. TRAF1-C5 as a risk locus for rheumatoid arthritis-a genomewide study. *New England Journal of Medicine*, 357(12):1199–1209, 2007.

[28] Soumya Raychaudhuri, Elaine F Remmers, Annette T Lee, Rachel Hackett, Candace Guiducci, Noël P Burtt, Lauren Gianniny, Benjamin D Korman, Leonid Padyukov, Fina AS Kurreeman, et al. Common variants at CD40 and other loci confer risk of rheumatoid arthritis. *Nature Genetics*, 40(10):1216, 2008.

[29] Daniel J Schaid, Wenan Chen, and Nicholas B Larson. From genome-wide associations to candidate causal variants by statistical fine-mapping. *Nature Reviews Genetics*, 19(8):491–504, 2018.

[30] Chang-Nam Son, So-Young Bang, Soo-Kyung Cho, Yoon-Kyoung Sung, Tae-Hwan Kim, Sang-Cheol Bae, and Jae-Bum Jun. The frequency of single nucleotide polymorphisms and their association with uric acid concentration based on data from genome-wide association studies in the Korean population. *Rheumatology International*, 34(6):777–783, 2014.

[31] Yaron Tomer, Lawrence M Dolan, George Kahaly, Jasmin Divers, Ralph B D'Agostino Jr, Giuseppina Imperatore, Dana Dabelea, Santica Marcovina, Mary Helen Black, Catherine Pihoker, et al. Genome wide identification of new genes and pathways in patients with both autoimmune thyroiditis and type 1 diabetes. *Journal of Autoimmunity*, 60:32–39, 2015.
